# Neural Correlates of Decision Thresholds in the Human Subthalamic Nucleus

**DOI:** 10.1016/j.cub.2016.01.051

**Published:** 2016-04-04

**Authors:** Damian M. Herz, Baltazar A. Zavala, Rafal Bogacz, Peter Brown

**Affiliations:** 1 Medical Research Council Brain Network Dynamics Unit at the University of Oxford, Mansfield Road, Oxford OX1 3TH, UK; 2 Nuffield Department of Clinical Neurosciences, University of Oxford, Level 6, West Wing, John Radcliffe Hospital, Oxford OX3 9DU, UK; 3 Surgical Neurology Branch, National Institutes of Health, 10 Center Drive, 3D 20, Bethesda, MD 20814, USA

## Abstract

If humans are faced with difficult choices when making decisions, the ability to slow down responses becomes critical in order to avoid suboptimal choices. Current models of decision making assume that the subthalamic nucleus (STN) mediates this function by elevating decision thresholds, thereby requiring more evidence to be accumulated before responding [1, 2, 3, 4, 5, 6, 7, 8, 9]. However, direct electrophysiological evidence for the exact role of STN during adjustment of decision thresholds is lacking. Here, we show that trial-by-trial variations in STN low-frequency oscillatory activity predict adjustments of decision thresholds before subjects make a response. The relationship between STN activity and decision thresholds critically depends on the subjects’ level of cautiousness. While increased oscillatory activity of the STN predicts elevated decision thresholds during high levels of cautiousness, it predicts decreased decision thresholds during low levels of cautiousness. This context-dependent relationship may be mediated by increased influence of the medial prefrontal cortex (mPFC)-STN pathway on decision thresholds during high cautiousness. Subjects who exhibit a stronger increase in phase alignment of low-frequency oscillatory activity in mPFC and STN before making a response have higher decision thresholds and commit fewer erroneous responses. Together, our results demonstrate that STN low-frequency oscillatory activity and corresponding mPFC-STN coupling are involved in determining how much evidence subjects accumulate before making a decision. This finding might explain why deep-brain stimulation of the STN can impair subjects’ ability to slow down responses and can induce impulsive suboptimal decisions.

## Highlights

• Subjects make a choice when the integrated evidence crosses a “decision threshold”
• Such thresholds are reflected by 2- to 8-Hz oscillations in the subthalamic nucleus
• The relationship between decision thresholds and STN activity is context dependent
• This is mediated by altered prefrontal-STN coupling during high cautiousness

## Teaser

In this article, Herz et al. show that decision thresholds in humans are modulated by subthalamic low-frequency oscillatory activity. This relationship depends on the subjects’ level of cautiousness, which is mediated by increased influence of the medial prefrontal cortex-subthalamic pathway on decision thresholds when caution is required.

## Results and Discussion

The main goal of this study was to test whether neural activity of the subthalamic nucleus (STN) is related to modulations of decision thresholds during perceptual decision making. This has been suggested by computational models of decision making [1, 4] and studies using fMRI [3, 6]. Here, we directly recorded STN local field potentials (LFPs) in Parkinson’s disease (PD) patients through electrodes implanted in the STN several days after deep-brain stimulation (DBS) surgery, while patients performed two versions of a moving dots task [10]. In both tasks, coherence rates of the moving dots linearly increased over time until 50% of all dots moved coherently in one direction. Participants pressed a button with their right or left index finger as soon as they perceived that the majority of dots were moving in the right or left direction. This design allowed us to assess neural activity, which is not related to abrupt stimulus changes or motor preparation, because changes in spectral STN activity were observed well before any choice was executed. Combining single-trial LFP analysis and drift diffusion modeling (DDM) allowed us to elucidate context-dependent relationships between single-trial oscillatory STN activity and features of decision making, which are not evident with conventional analyses of reaction times (RTs) and accuracy rates. For a detailed analysis of trial-averaged time frequency spectra related to the tasks, the reader is referred to previous reports by Zavala and colleagues [11, 12].

**Figure 1:**
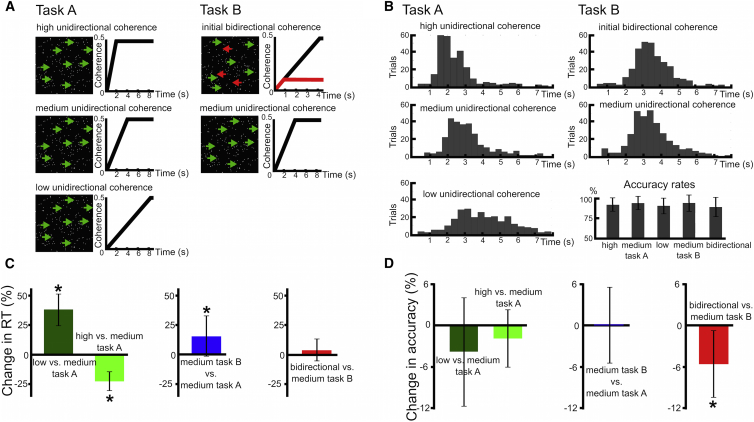
Experimental Tasks and Behavioral Analyses (A) Experimental tasks A and B. In task A (first column), the rate of coherently moving dots changed between conditions (low, medium, and high unidirectional coherence). Black traces illustrate how coherence changed over time in the different conditions. In task B, 50% of trials showed dots moving coherently in opposite directions until dots moving in the incorrect direction were capped (red trace in right upper panel), while the remaining 50% of trials were identical to medium unidirectional coherence trials in task A. (B) RT histograms and accuracy rates of all conditions are shown. (C and D) Effects of the experimental manipulations on RT and accuracy. Columns reflect delta values. Error bars indicate SD. Asterisks indicate significance at p_corrected_ 0.05.

In task A, differences in the rate at which dots increased coherence were used to alter the rate of sensory evidence accumulation (left column in Figure 1A). Trials with low unidirectional coherence had significantly higher RTs relative to trials with medium unidirectional coherence (mean RT increase 38.1% ± 13.5 SD, z_(10)_ = 2.934, P_corrected_ = 0.012). Conversely, trials with high unidirectional coherence had significantly lower RTs relative to medium unidirectional coherence (mean RT decrease 22.4% ± 8.1 SD, z_(10)_ = −2.934, P_corrected_ = 0.012). Changing coherence in task A did not affect accuracy rates (change in accuracy during low unidirectional coherence relative to medium unidirectional coherence −3.7% ± 7.7 SD, z_(10)_ = −1.481, P_corrected_ = 0.556; change in accuracy during high unidirectional coherence relative to medium unidirectional coherence −1.8% ± 4.1 SD, z_(10)_ = −1.680, P_corrected_ = 0.372), see Figures 1B–1D. In task B, in 50% of trials the number of dots moving coherently *both* to the right and left increased until 0.83 s, after which the dots moving into the incorrect direction no longer increased in coherence, while the dots moving into the correct direction further increased coherence (right column in Figure 1A). There was thus no relative evidence for either direction in the first 0.83 s, particularly as neural integrators are thought to integrate the difference in dot coherence [13]. The remaining trials in task B were identical to trials with medium unidirectional coherence in task A. However, RTs in these trials in task B were significantly higher compared to identical trials in task A (relative increase in RT: 15.7% ± 17.1 SD, z_(10)_ = 2.578, P_corrected_ = 0.040), while accuracy was similar (change in accuracy 0.1% ± 5.6 SD, z_(10)_ = −0.105, P_uncorrected_ = 0.917; Figures 1B–1D). This observation was in line with our a priori hypothesis that the presence of intermixed trials with initial bidirectional coherence in task B increased patients’ level of cautiousness. Thus, they accumulated more evidence before making a decision in trials with medium unidirectional coherence in task B compared to task A. Finally, in task B RTs were similar in trials with initial bidirectional coherence and trials with unidirectional medium coherence (difference in RT: 4.3% ± 9.4% SD, z_(10)_ = 1.511, P_corrected_ = 0.524), while accuracy significantly decreased by 5.6% ± 4.9% SD (z_(10)_ = −2.668, P_corrected_ = 0.032; Figures 1B–1D). This finding indicates that participants committed more erroneous responses in trials with initial bidirectional coherence when they did not accumulate sufficient evidence.

**Figure 2:**
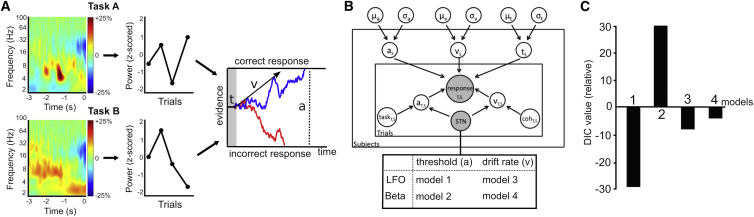
HDDM Analysis (A) The time frequency plots show a pre-response increase in LFO power (time 0 indicates the response) relative to baseline averaged across conditions in both tasks (first column). Single trial LFPs were Z-scored for each task separately before entering them into the HDDM (second column). In DDM, *t* is the non-decision time (e.g., related to afferent delays and motor execution), and *v* is the drift rate indicating the rate of evidence accumulation until threshold *a* is reached and the response is executed (third column). Blue and red traces are examples of a single correct and incorrect response, respectively. Please note that this is a schematic illustration and does not show the actual model parameters. (B) Illustration of HDDM. Parameters *a*, *v*, and *t* were estimated simultaneously for the group (circles outside the plates with group mean μ and variance σ) and subjects *S* (circles in outer plate). Variations in *a* and *v* were modulated by experimental manipulations (coh, coherence: trials with low and high unidirectional coherence and trials with initial bidirectional coherence relative to medium unidirectional coherence; task, task B relative to task A) at each trial *T* (circles in inner plate). Observed data are represented by shaded circles. They comprised responses (with RT and accuracy) and single-trial STN activity. The four neural HDDMs, which were compared, are shown in the box under the HDDM graphic. Please see Figure S1 for parameters of the HDDM without neural data and Figures S2 and S3 for validation of the HDDM. (C) Model comparison. DIC values are shown relative to DIC of the HDDM not containing any neural data. Relative DIC were −29 (model 1), +30 (model 2), −8 (model 3), and −4 (model 4).

In order to test whether the observed behavioral effects could be related to modulation of the rate of evidence accumulation and decision thresholds, we modeled these latent processes underlying the observed behavior in the drift diffusion framework [14]. In DDM, sensory evidence is accumulated over time until the integrated evidence crosses the decision threshold and the choice is executed (see third column in Figure 2A). We applied a hierarchical Bayesian estimation of DDM parameters (HDDM), which is particularly suited for studies with relatively few trials [15]. As expected from the behavioral results, changing the amount of coherently moving dots significantly modulated drift rates; i.e., drift rates were lower in trials with low unidirectional coherence and initial bidirectional coherence and higher in trials with high unidirectional coherence compared to trials with medium unidirectional coherence (100% posterior probability for all effects being different than 0). Including trials with initial bidirectional coherence in task B significantly elevated decision thresholds, i.e., thresholds were higher in task B compared to task A (100% posterior probability). Please see Figure S1 and Supplemental Experimental Procedures for more details. This model had much stronger evidence compared to models proposing only changes in drift rate (difference in deviance information criterion [DIC], 34) or threshold (difference in DIC 121) and adequately predicted the observed behavior (Figure S1). We additionally validated HDDM by applying a non-hierarchical DDM (NHDDM) to the data, which yielded highly similar model parameter estimates at the group and individual subject level (Figure S2), and by applying HDDM to a simulated dataset (Figure S3; see also Supplemental Experimental Procedures). The observation that participants did not have significantly longer RTs in trials with an initial bidirectional coherence compared to trials with medium unidirectional coherence in task B indicates that decision thresholds might have changed not only between tasks, but also between conditions in task B. Allowing thresholds to change between conditions in task B in HDDM showed that thresholds were higher in both conditions in task B compared to task A (> 99% posterior probability), but also higher in the medium unidirectional coherence trials in task B compared to trials with initial bidirectional coherence (>99% posterior probability). Nevertheless, as the main aim of this study was to investigate the role of the STN during perceptual decision making, we used a less complex, a-priori-defined model postulating thresholds adjustments between tasks, not conditions, below.

Accordingly, we assessed whether trial-by-trial measurements of STN activity—as reflected by LFP changes before the response—modulated different latent decision-making parameters at each trial using HDDM regression analysis. To this end, we computed single trial estimates of STN power in the time period preceding participants’ responses (from −3 s until the response) and Z-scored these values separately for task A and B before including them in the HDDM (Figures 2A and 2B). We then estimated and compared four neural HDDMs based on a-priori-defined hypotheses, which differed in the precise frequency range of STN-LFP activity (2–8 Hz low-frequency oscillations [LFOs] versus 13–30 Hz beta oscillations) and the latent variable, which was modified by STN activity (threshold versus drift rate). Of note, there was no significant overall difference in pre-response STN LFO power between task A and B (t = 1.646, p = 0.115). Allowing trial-by-trial STN-LFO to modulate threshold estimates in the HDDM significantly improved model evidence compared to the model not containing any neural data (difference in DIC 29), and also clearly outperformed the alternative neural HDDMs (Figure 2C). Thus, model selection provided strong evidence that trial-by-trial variations in decision thresholds are modulated by STN-LFO.

**Figure 3:**
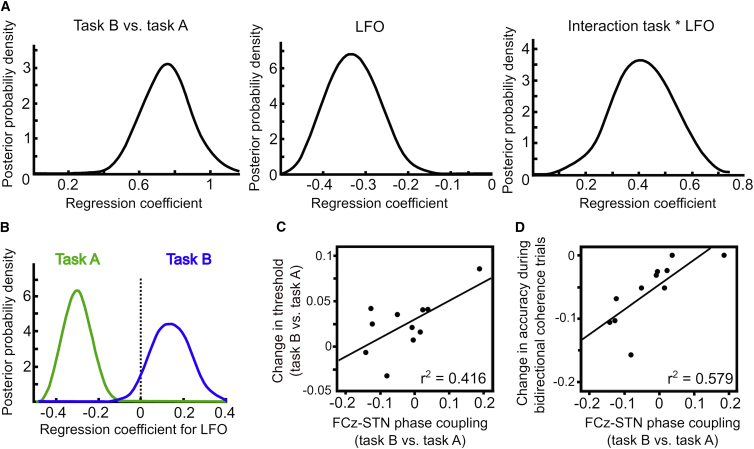
Neural Modulations of Decision Thresholds (A) Posterior probabilities for modulation of decision thresholds by task (task B relative to task A), LFO, and their interaction. Peaks reflect the best estimates, while width represents uncertainty. (B) Post hoc analysis showed an opposite relationship between LFO and thresholds for task A and B. (C) Second (group) level regression between change in FCz-STN coupling (task B versus task A) and adjustments of decision thresholds derived from NHDDM (p = 0.032). (D) Regression between change in FCz-STN coupling and participants’ ability to control erroneous responses during trials with initial bidirectional coherence (p = 0.007).

Next, we aimed to investigate the exact relationship between STN-LFO and decision thresholds. To this end, we analyzed how decision thresholds varied as a function of STN LFO during both tasks by inspecting the posterior probability distribution of model parameters. We found a significant main effect of task, a significant main effect of STN-LFO and, critically, a significant interaction between task and STN-LFO (100% posterior probability for all parameters being different than 0, see Figure 3A). This interaction indicates that the effect of STN-LFO on decision thresholds critically depends on the level of cautiousness, which was higher in task B (see above). These results did not change when using non-Z-scored single trial estimates of STN activity or when using different wavelet lengths for computing STN power (see Supplemental Experimental Procedures). Post hoc tests of the effect of STN-LFO in task A (low cautiousness) and task B (high cautiousness) revealed that high power of STN-LFO predicted decreased decision thresholds in task A (100% posterior probability), while it predicted elevated decision thresholds in task B (95% posterior probability; Figure 3B). This context-dependent relationship did not change when using a more complex model where thresholds could vary between all conditions. In this additional control analysis all significant regression coefficients were negative in task A (100% probability for trials with low and high unidirectional coherence) and positive in task B (100% probability for trials with initial bidirectional coherence).

These results indicate that STN activity, as reflected by LFO, does not simply reflect increases in decision thresholds, but that this relationship critically depends on the level of cautiousness. A possible explanation for this observation is a flexible reorganization of cortico-STN networks depending on task demands enabling the medial prefrontal cortex (mPFC) to increase its influence over STN function [2, 3, 4, 7, 9, 11, 16]. To test this hypothesis, we analyzed connectivity between electroencephalography (EEG) electrode FCz and STN by computing the inter-site-phase clustering (IPC) (see Supplemental Experimental Procedures) reflecting how reliably the phases of oscillations in FCz and STN were aligned prior to the response. We then tested whether the extent to which IPC changed between task A and B predicted how much participants adjusted their decision thresholds estimated using NHDDM. This analysis showed that while there were no overall changes in FCz-STN IPC between tasks (z_(10)_ = 1.067, p = 0.286) the extent to which participants increased FCz-STN IPC significantly predicted adjustments in decision thresholds (r^2^ = 0.416, p = 0.032), see Figure 3C. Furthermore, adjustments in FCz-STN IPC also predicted participants’ ability to control erroneous responses (r^2^ = 0.579, p = 0.007), see Figure 3D. Of note, these results stayed significant even when accounting for individual differences in drift rates (thresholds: r^2^ = 0.413, p = 0.045; accuracy: r^2^ = 0.601, p = 0.008). These results suggest that mPFC-STN communication through phase alignment might be an important mechanism for adjusting decision thresholds and thereby controlling erroneous responses when participants are more careful in making decisions, although it should be noted that regression analyses were based on relatively few observations (n = 11).

In conclusion, we report three novel findings in this study. First, our results demonstrate for the first time that oscillatory STN activity reflects trial-by-trial modulations of decision thresholds, i.e., how much evidence subjects integrate before making a decision. This relationship is specific for the latent mechanism underlying decision making (thresholds, but not drift rates) and frequency range of oscillatory activity (LFO, but not beta oscillations). Second, we show that STN activity does not uni-directionally increase decision thresholds but can have opposing effects on thresholds depending on subjects’ level of cautiousness. Finally, we found that modulations of the phase alignment between mPFC and STN, a mechanism that might optimize information transfer between these two regions [17], predicts adjustments of decision thresholds and participants ability to control erroneous responses. Thus, a context-dependent integration of STN in dynamic cortico-STN networks might be critical in the ability to adjust behavior to changing environments and give rise to the context-specific relationships between STN activity and modulation of decision thresholds observed in this study. This neural mechanism might be affected in individuals who express impulsive behavior during therapeutic stimulation of the STN [2, 4, 5]. It remains to be elucidated whether such unwanted effects of DBS can be avoided by specifically targeting abnormal (beta) oscillations in PD [18] leaving modulations of LFO relatively intact.

## Author Contributions

B.A.Z. and P.B. designed the experiments. D.M.H. and R.B. conducted the drift diffusion modeling analysis. D.M.H. and B.A.Z. conducted the electrophysiological analysis. P.B. supervised the project. D.M.H. wrote the first draft of the manuscript. B.A.Z., R.B., and P.B. contributed to editing and revising the manuscript.

## Supplemental Information

Document S1. Figures S1–S3, Table S1, and Supplemental Experimental Procedures

Document S2. Article plus Supplemental Information
