# Supplementary material for: Neural Correlates of Decision Thresholds in the Human Subthalamic Nucleus
Source: Curr Biol. 2016 Apr 4;26(7):916–20. doi: 10.1016/j.cub.2016.01.051 (PMC4826434; doi:10.1016/j.cub.2016.01.051)
Supplement: Document S1. Figures S1–S3, Table S1, and Supplemental Experimental Procedures [file mmc1.pdf]

Current Biology, Volume *26*

Supplemental Information

Neural Correlates of Decision Thresholds in the Human Subthalamic Nucleus

Damian M. Herz, Baltazar A. Zavala, Rafal Bogacz, and Peter Brown

Supplemental information

Supplemental figures:

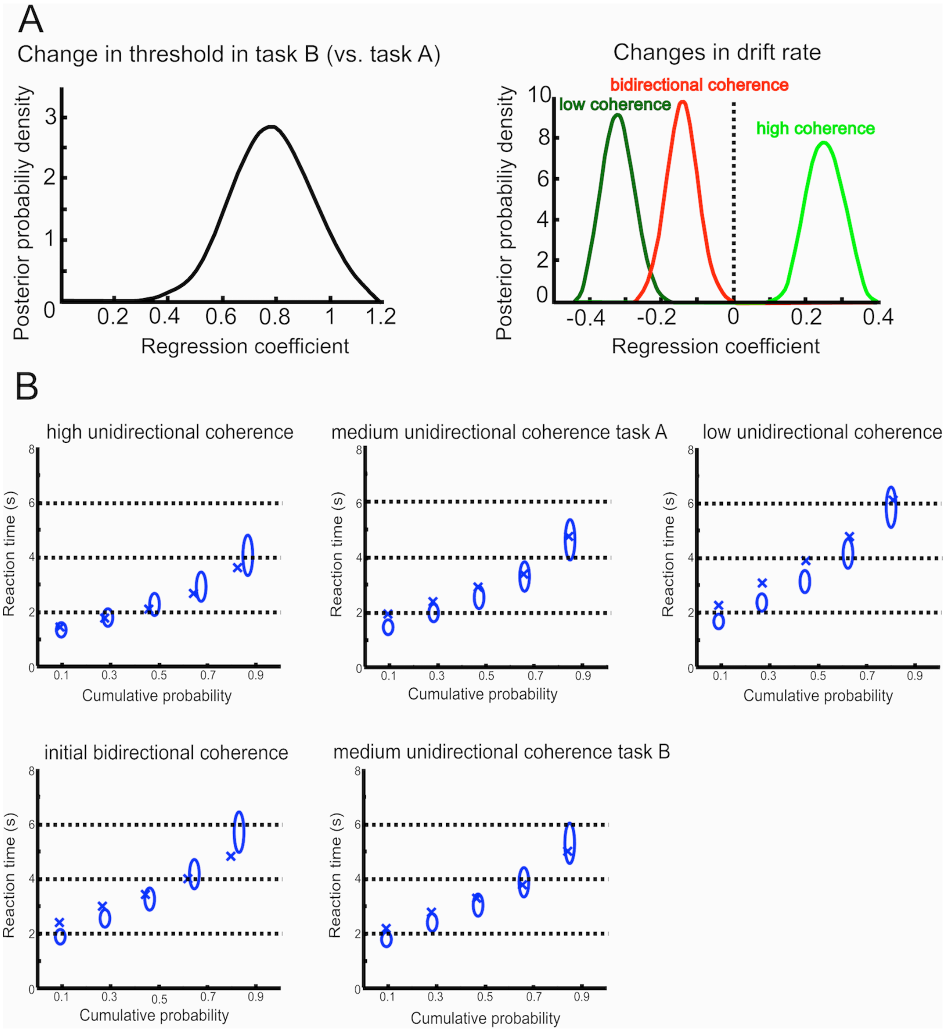

Figure S1: HDDM without neural data. Related to figure 2. (A) Main effects on decision thresholds and drift rates of the HDDM not containing any neural data. The left panel shows the posterior distribution of the regression coefficient for the effects on the decision thresholds. The right panel shows the posterior distribution of coefficients for the effects on drift rates. Peaks reflect the best parameter estimate, while the width of the distribution represents uncertainty. All posterior distributions are shifted away from zero (i.e. they do not overlap with 0 on the x-axis) and thus have 100% posterior probability to be different than 0. Note that threshold regression coefficients (left panel) are shown relative to task A, i.e. the threshold estimate during task A constitutes the intersect in the regression. Similarly, drift rate regression coefficients (right panel) are shown relative to drift rate during medium coherence (see also Supplemental Experimental Procedures). (B) Quantile probability plots. Observed mean reaction times (RT) for five quantiles (10, 30, 50, 70 and 90 %) are marked by a cross and plotted against their cumulative probability for the five conditions separately. Predicted quantile mean RTs are marked as ellipses with the width capturing estimation uncertainty (standard deviation of the posterior predictive distribution from the model). The plot shows that the HDDM provided overall good predictions of the observed data and, more importantly, captured effects of task manipulations on RT. For example, it predicts an RT shift upwards from high to low unidirectional coherence trials as well as increased RT in medium unidirectional coherence trials in task B compared to medium unidirectional coherence trials in task A. Due to the low number of error trials quantile probability plots are only shown for correct trials. Nevertheless, predicted error rates can be evaluated by the predicted cumulative probabilities along the x-axis (e.g. poor predictions of error rates would results in incongruencies between predicted and observed quantile RT along the x-axis).

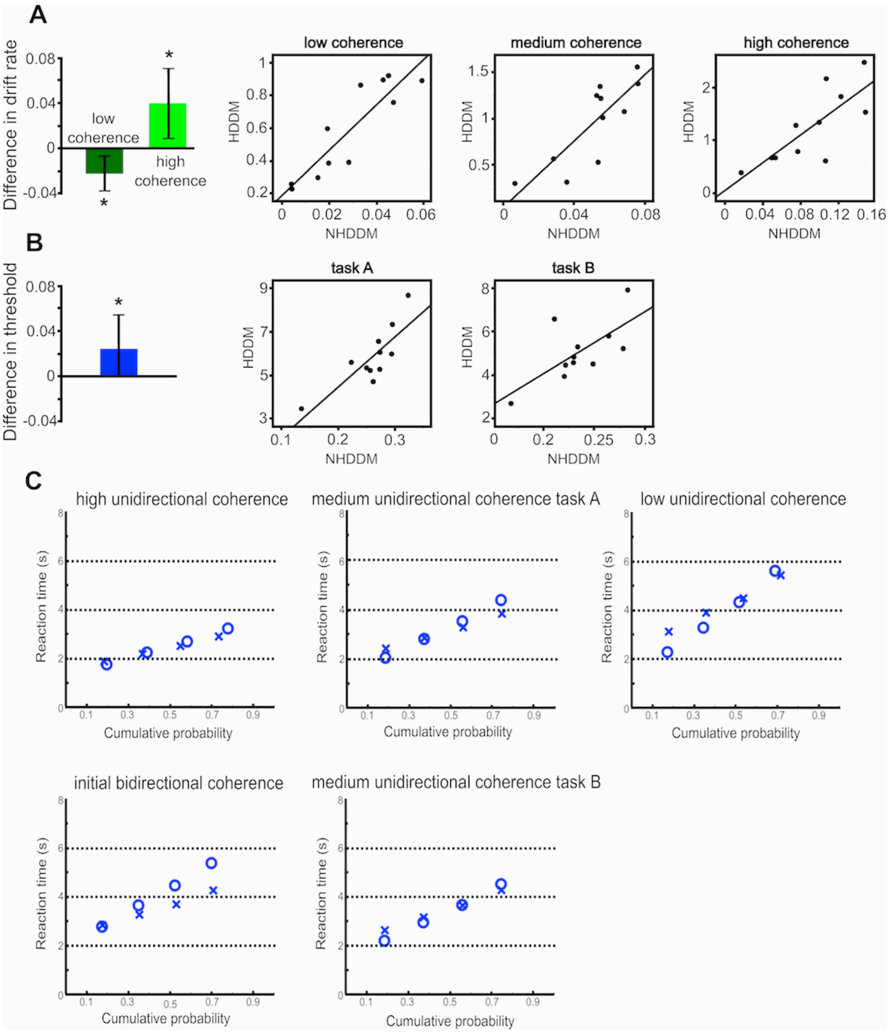

Figure S2: Non-hierarchical DDM (NHDDM). Related to figure 2. (A) Drift rate slope parameters estimated from the NHDDM showed a significant difference in drift rate slopes in low compared to medium unidirectional coherence trials (-0.022 ± 0.015 SD, $z_{(10)} = -2.756$, $P_{corrected} = 0.012$) and high compared to medium unidirectional coherence trials (0.040 ± 0.031 SD, $z_{(10)} = 2.845$, $P_{corrected} = 0.008$; Wilcoxon signed rank tests). Thus, the mean effects of coherence manipulations on drift rates were similar to the ones obtained from the HDDM (figure S1). Note that in the NHDDM there was no separate drift rate slope for trials with initial bidirectional coherence, since these drift rate slopes were modeled as medium drift rate slopes with an 0.83 s delay according to the delayed onset of relative increase in evidence accumulation in this condition. The correlation plots show that drift rate parameters estimated from NHDDM and HDDM were also proportionally similar, i.e. participants with high drift rate slopes in NHDDM had high drift rates in the HDDM and vice versa (low coherence: rho 0.892, $P_{corrected} = 0.003$, medium coherence: rho 0.824, $P_{corrected} = 0.006$, high coherence: rho 0.785, $P_{corrected} = 0.012$; Pearson correlations). (B) Like the HDDM, NHDDM threshold parameters showed a significant increase in threshold in task B compared to task A (0.024 ± 0.031 SD, $z_{(10)} = 2.223$, $P = 0.026$; Wilcoxon signed rank test). Furthermore, threshold parameters were also proportionally similar as indicated by correlation analyses (task A: rho 0.822, $P_{corrected} = 0.004$, task B: rho 0.682, $P_{corrected} = 0.042$, Pearson correlations). Note that parameters estimated in HDDM are scaled with a factor of 10 compared to 'traditional' DDM values (e.g. a drift rate of 0.1 in the NHDDM corresponds to a drift rate of 1 in HDDM). (C) Quantile probability plots. Observed mean RTs for four quantiles (20, 40, 60 and 80 %) are marked by a cross and plotted against their cumulative probability for the five conditions separately. Predicted quantile mean RTs are marked as circles. The plot shows that the NHDDM, like the HDDM, provided overall good predictions of the observed data and captured effects of task manipulations on RT (see also figure S1).

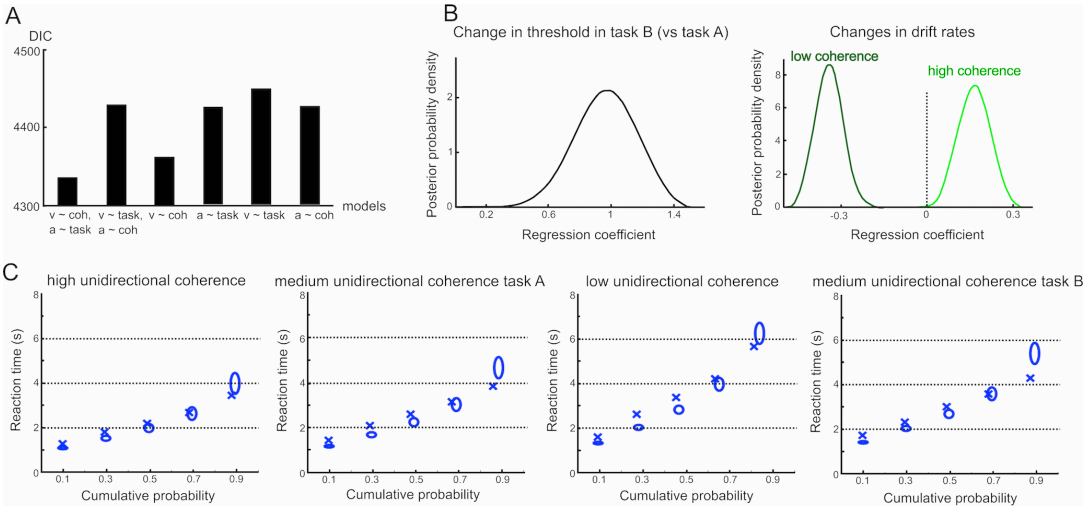

Figure S3: Results of the simulation analysis. Related to figure 2. A dataset (RT and accuracy) was simulated based on threshold and drift rate slope parameters of a representative patient (patient with parameters closest to the group mean). HDDM was the fitted to this simulated dataset. (A) We compared different models, which differed in the decisiosn parameter, which was modulated during the tasks (drift rate and threshold, only drift rate or only threshold) and the experimental manipulation, which modulated these latent parameters (coherence and task, only coherence and only task). The true model, which was used for simulating the dataset, consisted of a drift rate slope modulation by coherence rate and a threshold modulation by task. This model was also clearly the best model according to its DIC (first column in (A); DIC difference to next best model 27), i.e. HDDM model evidence favored the true model over the alternative models. DIC values for all models were: model S1 (a ~ task, v ~ coh) 4336, model S2 (v ~ task, a ~ coh) 4429, model S3 (v ~ coh) 4363, model S4 (a ~ task) 4426, model S5 (v ~ task) 4449, model S6 (a ~ coh) 4427. The recovered parameters are shown in (B), with a significant change in threshold in task B vs. task A and significant changes in drift rates in trials with low and high unidirectional coherence compared to trials with medium unidirectional coherence (100% posterior probability for all parameter estimates being different than 0). Interestingly, the model was also able to recover that the true drift rate manipulation during low unidirectional coherence was stronger than the change in drift rate during high unidirectional coherence (relative change in the drift rate slope used for simulation was -0.028 for low unidirectional coherence and +0.021 for high unidirectional coherence). (C) shows quantile probability plots with observed (cross) and predicted (ellipses) RT distribution for the four considered conditions. As in figure S1, the width of ellipses indicates estimation uncertainty (standard deviation).

Supplemental tables:

Patient	Age	Disease duration	UPDRS-III OFF	UPDRS-III ON	First symptom	Reason for surgery	Medication
1	49	10	42	6	Tremor	Tremor	Levodopa 300; Trihexyphenidyl 2
2	50	4	N/A	N/A	Tremor	Tremor	Levodopa 400; Rotigotine 16; Entacapone 600
3	66	16	32	13	Loss of dexterity	Bradykinesia	Levodopa 600; Ropinirole 24; Rasagiline 1; Amantadine 200;
4	51	7	58	13	Loss of sense of smell	Tremor, gait difficulties	Levodopa 1300
5	64	12	70	20	Tremor	Dyskinesias	Levodopa 1200; Apomorphine 7 mg/h
6	47	14	34	11	Bradykinesia	Dyskinesias, motor fluctuations	Levodopa 350; Pramipexole 1.05; Amantadine 300
7	66	14	63	24	Shoulder pain, stiffness	Motor fluctuations	Levodopa 650; Pergolide 9
8	57	6	21	7	Bradykinesia	Dyskinesias, motor fluctuations	Levodopa 750; Entacapone 1000
9	61	4	37	15	Tremor	Tremor	Levodopa 750; Amantadine 200; Entacapone 1000
10	65	15	51	21	Tremor	Freezing	Levodopa 400; Ropinirole 12; Amantadine 200
11	42	9	60	42	Loss of dexterity	Bradykinesia, dystonia, freezing	Levodopa 600; Amantadine 400
mean	56.2	10.1	46.8	17.2			

Table S1: Clinical and demographic characteristics of included patients. Age is given in years, medication is given in mg/day unless otherwise stated. N/A, not available.

Supplemental Experimental Procedures:

Participants

Eleven patients (six males, mean age 56.2 years ± 8.7 (standard deviation, SD), mean disease duration 10.1 years ± 4.4 SD) with Parkinson's disease (PD) undergoing deep brain stimulation (DBS) of the subthalamic nucleus (STN) were enrolled in the study (for clinical details see table S1). In all patients a quadripolar macroelectrode (model 3389, Medtronic Neurologic Division, Minneapolis, MN, USA) featuring four platinum-iridium cylindrical surfaces was implanted in bilateral STN. Lead localization was confirmed either by intra-operative stereotactic magnetic resonance imaging or by the clinical effect during the operation and immediate post-operative stereotactic computerized tomography. DBS electrode extension cables were externalized enabling recordings prior to implantation of a subcutaneous pacemaker up to seven days later.

A healthy control group could not be included in the study due to the invasive nature of electrode implementation. However, in order to approximate physiological function of STN as closely as possible, all recordings were conducted in patients on their normal medication. Importantly, the dopaminergic state in PD does not affect decision thresholds [S1], which were the main focus of this study. Mean improvement in motor function as indexed by Unified Parkinson's Disease Rating Scale-III was 63.9 % ± 14.8 SD after a levodopa challenge indicating a very good dopamine response in the studied patient group. All patients completed the two experimental tasks consecutively on the same day, three to six days after electrode implantation. In accordance with the Declaration of Helsinki, all

patients gave their written informed consent to participate in the study, which was approved by the local ethics committee. Of note, data of the same patient group has been reported previously concerning separate group analysis of averaged time frequency spectra during the tasks [S2, S3]. Here, for the first time, we demonstrate that trial-by-trial variations of STN activity (which are considered noise in averaged time-frequency spectra) predict context-dependent adaptations of decision-thresholds during perceptual decision-making.

Paradigms

The experimental tasks have been described in detail previously [S2, S3] and are illustrated in Fig. 1A. A cloud of 200 randomly moving white dots was presented on a black background. The tasks were presented on a 33 cm Macintosh laptop with a 60 Hz screen refresh rate using PsychoPy [S4]. The cloud was 14 cm in diameter and dots were 10 pixels large (visual angle ≈ 0.25°). Each randomly moving dot moved in a straight line at a rate of 0.14 mm per frame for 20 frames (333 ms) before moving to another part of the cloud and moving in a new direction chosen pseudorandomly between -180° and 180°. Participants were instructed to indicate the overall direction of dots whenever they noticed that the cloud was moving to the left or to the right side. They were instructed to respond as fast and accurately as possible, i.e. to balance speed and accuracy. Choices were indicated by a left ("z") or right ("/") button press on a keyboard with the left and right index finger, respectively. Participants performed task A first. The task comprised three randomly interspersed conditions. During trials with high unidirectional coherence the number of dots moving coherently into either the left or right side of the screen increased linearly from 0% to 50% within 2.083 s (corresponding to 0.004% per frame). During trials with medium and low unidirectional coherence 50% coherence was reached after 4.17 s (0.002% increase per frame) and 8.333 (0.001% increase per frame), respectively. In task B, participants were told that in some of the trials dots would start moving in opposite directions instead of just one direction. In these trials the number of dots moving coherently both to the right and left increased at the same rate until 0.83 s. After 0.83 s, the dots moving in the incorrect direction no longer increased in coherence (i.e. they were capped at 10% of all 200 dots), while the dots moving into the correct direction further increased their coherent motion until reaching 50% coherence after 4.17s. The remaining trials of task B were identical to trials with medium unidirectional coherence in task A (i.e. the number of dots moving coherently in one direction increased linearly from 0% to 50% within 4.17s). In both tasks, all trials were pseudo-randomly presented with equal probability (28 of 84 trials per condition in task A, 40 of 80 trials per condition in task B). Visual feedback ("incorrect") was provided for a duration of 0.75 s only in case of erroneous responses or prolonged response times > 14 s. Before each trial, dots moved randomly for a time period between 2 and 4 s. Prior to commencement of recordings participants could practice the tasks as long as they wished (usually < 10 trials).

We chose to use coherence rates, which linearly increased over time in contrast to time-invariant coherence differences between conditions in order to avoid presenting subjects with an explicit cue in the beginning of each trial. Thus, at no point subjects were given a 'cue' indicating that the trial had begun. After the subject made the response for a given trial, all dots immediately began to move in random direction for a duration of 2 - 4 s before slowing starting to move coherently again for the next trial. This design allowed us to assess neural activity, which is not related to abrupt stimulus changes or motor preparation, because the changes in spectral STN activity were observed well before the choice was executed [S2, S3].

Analysis of behavioral data

All trials with RT > 8 s or < 0.25 s were discarded from further analyses. There were three main experimental manipulations during the tasks. First, an increase (high unidirectional coherence) or decrease (low unidirectional coherence) in the rate of coherently moving dots relative to trials with medium unidirectional coherence in task A. Second, the difference between identical trials with medium unidirectional coherence rates in task B and task A, which only differed regarding the presence of intermixed trials with initial bidirectional coherence in task B. We hypothesized that the presence of such trials would increase participants' level of cautiousness due to the increased task demands. Third, the difference between trials with initial bidirectional coherence and medium unidirectional coherence trials in task B. The first 0.83s in the former trials did not convey any relative evidence for either direction.

To assess effects on reaction times (RT) and accuracy rates we computed the relative change in RT (e.g. (RT during high unidirectional coherence – RT during medium unidirectional coherence) / RT during medium unidirectional coherence) and accuracy rates (e.g. (Accuracy during high unidirectional coherence – Accuracy during medium unidirectional coherence) / Accuracy during medium unidirectional coherence) and used non-parametric one-sample Wilcoxon signed-rank tests to test the

resulting values against a median of 0 using SPSS statistics (v22, IBM, New York, USA). The significance threshold was set to 0.05 adjusting for multiple comparisons using the Bonferroni method.

Analysis of trial-by-trial LFPs

Local field potentials (LFP) were recorded from DBS electrodes. In addition, electroencephalography (EEG) was recorded from FCz, Cz and Pz according to the international 10-20 system. A wider coverage with EEG electrodes was not possible due to surgical wounds and dressings. Preprocessing of electrophysiological data was identical to the procedures reported previously [S2, S3]. In short, all signals were sampled at 2048 Hz, band-pass filtered between 0.5 and 500 Hz, amplified (TMSi porti, TMS International, Enschede, The Netherlands), down-sampled to 1000 Hz and notch filtered at 50 Hz. LFPs were then converted to a bipolar montage between contacts (three channels per STN) and EEG electrodes were re-referenced to Cz to limit effects of volume conduction. Trials with clear artifacts were discarded. After removal of trials with artifacts and behavioral outliers, 29.5 trials per subject and condition and 1621 trials in total remained. Power and phase of LFPs were computed using the continuous wavelet transform (1 Hz frequency resolution, 10 cycles per frequency) and resulting time frequency spectra were chunked into individual trials based on the onset of the motor responses for each task separately. To control that the relatively long wavelets did not lead to smearing of LFP power across trials, we repeated this analysis using 5 cycle wavelets. Single trial power estimates of low frequency oscillations (LFO, see below) using 10 cycle wavelets and 5 cycle wavelets, respectively, were highly correlated (rho = 0.95, Spearman correlation over all trials) and yielded identical results in the HDDM analysis (see below; data is only shown for the analysis using 10 cycle wavelets). Since group analysis of time-frequency spectra during the tasks showed a sustained pre-response increase in LFO from approximately 3 s prior to the button presses [S2, S3], see also fig. 2A, power values were extracted from -3 to 0 seconds. In case response time in a given trial was < 3 s (median RT was 3.113 s) time windows for extracting single-trial pre-response power values were restricted to –RT to 0 in order to avoid modeling STN activity prior to trial onset. Single trial estimates were then averaged across the respective time window and frequencies (2-8 Hz and 13-30 Hz, see below) and normalized to the mean power of those frequencies in the peri-response window (-3 to +2 s) of each participant and finally averaged across all STN electrodes. We chose to average across STN electrodes in order to avoid selection bias, even though this procedure might underestimate spectral changes. Importantly, neither STN -power, nor -FCz connectivity values differed regarding localization on the dorso-ventral STN axis [S2]. The 2-8 Hz window was chosen, because increases in LFO and cortico-STN connectivity in the current tasks did not have a clear lower boundary at 4 Hz (theta) [S2, S3], which is in agreement with previously reported motor conflict-related power changes in STN [S5, S6]. Beta power (13-30 Hz) was included, because of its central role in motor processing in the STN [S7]. To assess whether there were overall differences in pre-response LFO power between task A and B we compared power values (averaged over the 2-8 Hz frequency window and fixed -3 to 0s time window) using paired samples t-test.

Drift diffusion modeling

The goal of drift diffusion modeling (DDM) in this study, was to investigate whether trial-by-trial fluctuations in pre-response STN power were related to modulations of latent processes underlying perceptual decision making and whether this relationship was specific to the frequency range of oscillatory activity (LFO vs. Beta) and decision-making process (decision threshold vs. drift rate). A Bayesian hierarchical DDM (HDDM) has been developed and optimized for such purposes allowing regression of trial-by-trial variations in brain activity on decision parameters [S8]. Importantly this toolbox is also particularly suited for studies with low trial counts [S5, S8, S9], such as the current study with restrictions on the possible duration of the experiment due to vulnerability of the studied patient group. In DDM choices between two alternative options (here pressing the left or right button) are simulated by a noisy process in which evidence is accumulated over time (here the fraction of dots moving into different directions) until the evidence for one choice over the other is sufficient and the response is executed. There are two main parameters of interest in DDM. First, the rate of evidence accumulation is reflected by the drift rate v, which critically depends on the presented stimulus. Thus, in the current tasks drift rate was assumed to be modulated by the rate of change in coherently moving dots (manipulations in task A) and a delay in relative evidence accumulation due to dots moving into opposite directions (manipulation in task B). Second, the decision threshold a determines how much evidence has to be accumulated until a decision is made and thus reflects a measure of cautiousness. In this study, this parameter was assumed to be modulated by the presence of intermixed trials with an initial bidirectional coherence in task B, i.e. task B vs. task A (see also results of the behavioral analysis). A third parameter is the non-decision time t reflecting processes unrelated to the decision

(e.g. sensory processing in visual areas and motor execution). These parameters are estimated by the model based on the observed behavior, i.e. accuracy and response times. DDM has been shown to reliably detect these latent decision-making parameters throughout a variety of different behavioral and neurophysiological experiments over the last few decades [S10]. In the recently developed HDDM the trade-off between random and fixed effects models is optimized by assuming that parameters from individual subjects are drawn from the group distribution while allowing variation from this distribution given sufficient evidence to overwhelm the group prior [S8]. Parameters for each subject and condition were modelled according to a normal (real valued parameters) or Gamma distribution (positive valued parameters) centered on the group mean with group variance. Prior distributions were informed by 23 previous studies reporting parameters on a range of decision-making tasks [S8]. The starting parameter z (often referred to as bias parameter) was fixed to 0.5, because dots moving to the right or left had equal probability, i.e. participants were not biased toward right or left responses in the experimental tasks. Markov chain Monte Carlo sampling methods were used for accurate Bayesian approximation of the posterior distribution of parameters (generating 20 000 samples, discarding 10 000 samples as burn-in and keeping every fifth sample). We inspected traces of model parameters, their autocorrelation and computed the R-hat (Gelman-Rubin) convergence statistics to ensure that the models had properly converged [S8].

First we assessed basic assumptions of the model without including any neural data. This comprised modulations of drift rate by differences in the rate of coherence, where we a-priori expected a decrease in drift rate in trials with low unidirectional coherence and trials with an initial bidirectional coherence and an increase in drift rate in trials with high unidirectional coherence relative to trials with medium unidirectional coherence as well as modulations of threshold by task with an increased threshold in task B relative to task A. We validated our model assumptions (both drift rate *and* threshold were modulated by the experimental manipulations) by testing that this model had stronger evidence than models postulating only a change in drift rate or threshold respectively, using the deviance information criterion (DIC). DIC is widely used for comparisons of hierarchical models where other measures (e.g. Bayesian information criterion) are not appropriate [S8, S9]. A lower DIC value for a given model (for the whole group) indicates higher likelihood for that model compared to an alternative model taking into account model complexity (degrees of freedom). Usually a DIC difference of 10 is considered significant [S11]. Parameters of the best model were analyzed by Bayesian hypothesis testing, i.e. the percentage of samples drawn from the posterior that fall within a certain region (e.g. > 0). Posterior probabilities ≥ 95% were considered significant. Please note that this value is not equivalent to p-values estimated by frequentist methods (e.g. Wilcoxon signed rank tests during the analysis of behavioral data), but it can be interpreted in a similar manner. In the article, we refer to these estimates as *posterior probabilities* to clearly demarcate them from p-values. To assess model predictions we computed quantile probability plots, a popular measure for assessing model performance in the DDM framework [S10]. Here, observed and predicted RT for the 10, 30, 50, 70 and 90 percentile of trials (e.g. 10% fastest trials, 30% fastest trials, etc.) were plotted against their observed and predicted cumulative probability for each condition. Due to the low number of error trials, we only plotted quantiles for correct trials (see figure S1).

Of note, we made two simplifying assumptions when using HDDM. First, we did not explicitly model that a relative difference in coherence (i.e. to the left or right side) first started after 0.83 s in 50% of trials in task B, but just assumed a different drift rate during these trials. Furthermore, we assumed that changing the rate of dot coherence over time would have a similar effect on evidence accumulation as a difference in dots coherence, which is constant over time. This assumption was supported by the observation that participants executed responses during equal levels of integrated evidence and not instantaneous evidence in task A [S3]. To validate this approach we also applied a non-hierarchical DDM using custom-written scripts in matlab (R2015a, The MathWorks, Natick, MA, USA), in which we specified the exact properties of the task. In particular this model assumed that drift rate in condition i was a linearly increasing function $v_i(t)=s_i t$, where s_i is the slope parameter for condition i, and t is the time from onset of coherent motion, or the time from which coherence of dots moving in the correct direction was higher than the coherence of dots moving in the other direction on trials with an initial bidirectional coherence. We applied a previously validated method for non-hierarchical estimation of model parameters using a subplex algorithm for minimizing the cost function defined as the difference between observed and predicted quantile RT distribution (20, 40, 60 and 80 percentile) according to a least-squares estimation [S12]. The parameters comprised drift rate slopes s_1 (trials with low unidirectional coherence in task A), s_2 (trials with medium unidirectional coherence in task A and B including a delayed onset of 0.83 s for trials with an initial bidirectional coherence in task B) and s_3 (trials with high unidirectional coherence in task A), threshold a_1 (task A) and a_2 (task B) as well as the non-decision time t. The parameters were fitted to the observed data (10 iterations of

random search for starting values, 70 optimization iterations, 50 tuning iterations and 20 repetitions of the whole process; please see Bogacz and Cohen [S12] for more details of the method), for each participant separately and the best model (least error) was used for inference on model parameters. The code can be made available upon request. This non-hierarchical DDM (NHDDM) allowed us to validate the HDDM method described above by specifying exact experimental manipulations (e.g. slope in drift rate) and furthermore allowed second (group) level regression analyses with neural parameters, which cannot be computed at the single trial level (inter-site-phase-clustering; see below). We compared results of the HDDM and NHDDM by assessing parameter estimates at the group and individual subject level and assessed NHDDM fits by computing quantile probability plots (figure S2).

Finally, we validated the HDDM by fitting it to a simulated dataset. The simulation was based on estimated parameters derived from NHDDM for a representative patient (patient with parameters which were closest to the group mean). We simulated a dataset consisting of 300 trials per condition based on the number of trials for the whole group. We used conditions with low, medium and high unidirectional coherence in task A and medium unidirectional coherence in task B (i.e. 1200 trials) in order to model a manipulation of drift rate slope (low and high relative to medium unidirectional coherence) and threshold (medium unidirectional coherence in task B relative to medium unidirectional coherence in task A). We then assessed whether HDDM could successfully recover the true condition-specific manipulations of parameters based on the simulated dataset. To this end, we computed model evidence (DIC) for the true model compared to alternative models comprising different possible combinations of drift rate and threshold modulations during the different conditions. Furthermore, we assessed whether the true parameter manipulations could be recovered (effects on drift rate and threshold) and compared the predicted with the observed RT distribution. This analysis showed that HDDM successfully recovered the true parameter manipulations, which is shown in figure S3.

After verifying assumptions of the HDDM applied in this study, we then entered trial-by-trial STN-LFPs (z-scored) into the HDDM to test whether fluctuations in STN activity modulated decision-making parameters. We z-scored single trial estimates of STN power by subtracting the mean and dividing by the standard deviation separately for task A and task B. To further assess intercept issues, we repeated the HDDM analysis using non-z-scored data, which yielded identical results. Thus, regression coefficients between STN-LFPs and the decision parameter were estimated within the same hierarchical model, which was used to estimate the parameters themselves. For example, the model postulating that decision threshold a on trial x was not only modulated by Task but also STN-LFO (and their interaction) was defined by the regression: $a(x) = \beta_0 + \beta_1 Task(x) + \beta_2 LFO(x) + \beta_3 Task(x)*LFO(x)$ Regressing across trials allowed inferences on the extent to which threshold changes with STN activity [S8]. We created four a-priori defined models which differed regarding the frequency band of STN power (LFO vs. Beta) and the decision parameter which was modulated (threshold vs. drift rate), see Fig. 2B. These models were then compared using their DIC (relative to the model not containing any neural data). The best model was used for inferences on model parameters using Bayesian hypothesis testing, i.e. according to their posterior probability densities (see above). Posteriors of regression coefficients for trial-wise regressors were estimated only at the group level to address potential collinearity among model parameters, for regularizing parameter estimates and to prevent parameter explosion [S8, S9].

Analysis of FCz-STN inter-site-phase-clustering and regression analyses

Models of basal ganglia function during decision-making postulate that decision thresholds can be adjusted through activation of a connection between mPFC and STN in case of choice uncertainty or decision conflict [S2, S5, S9, S13-15]. To test this hypothesis, we aimed to assess whether mPFC-STN connectivity predicted adjustment of decision-thresholds and whether this relationship differed between low and high levels of cautiousness. However, connectivity parameters are more robust when averaged across trials compared to single-trial parameter estimates. Therefore, we computed estimates of inter-site-phase-clustering (IPC), a phase-based measure of connectivity, between FCz and STN for each subject and task and tested whether this measure predicted inter-individual differences in decision-thresholds. We only analyzed IPC between FCz and STN, because our previous studies have shown that task-related changes in cortico-STN connectivity were specific for the FCz-STN connection and were not observed between Pz and STN [S2, S3]. Please note that while we cannot discount more lateral prefrontal areas contributing to the signal at FCz due to the limited coverage in this study, previous studies have provided converging evidences that conflict-related LFO at FCz are generated in the mPFC [S5, S9, S16]. IPC was used as a measure of the extent to which oscillations in the mPFC and STN were phase-locked before the response [S2]. Analysis was done separately for task A and task B. Raw data were band-pass filtered between 2 and 8 Hz, and power and phase were computed for each STN channel and the FCz recording using the Hilbert transform. Then the magnitude of the average

phase difference between the STN-LFP and EEG signal were calculated at each time point, averaged across trials and a sliding window (1.6 s) was applied for integrating over time [S2]. For specifically computing the pre-response change in IPC values were averaged across the time window from -3 to 0 and divided by the mean peri-response (-5 to + 2s) value. This was done for each STN-channel and FCz recording separately and then averaged across STN channels for each participant resulting in one IPC value for each patient and task, which was used for regression analyses.

In order to test whether inter-individual differences in modulation of IPC between FCz and STN predicted differences in adjustments of decision thresholds we applied linear regression analyses. We used the difference in FCz-STN IPC between task B and task A (positive values indicating increased phase coupling) as predictor and the corresponding change in threshold estimates derived from the NHDDM as dependent variable. Since elevated decision-thresholds are thought to improve the ability to control erroneous responses, a second regression with the same predictor was conducted using the change in accuracy during trials with an initial bidirectional coherence (see results) as dependent variable. To account for possible confounding effects of differences in drift rate we additionally repeated these regression analyses partialing out drift rate estimates (partial regression). Note that we did not use the parameter estimates from the HDDM, because the hierarchical design violates the assumption of independence of observations. Prior to conducting regression analyses we ensured that assumptions of linear regression were not violated including independence of observations, lack of outliers (> 3*SD), and approximate normal distribution of residuals. The significance threshold for was set to 0.05 two-tailed.

Supplemental References:

S1. Huang, Y.T., Georgiev, D., Foltynie, T., Limousin, P., Speekenbrink, M., and Jahanshahi, M. (2015). Different effects of dopaminergic medication on perceptual decision-making in Parkinson's disease as a function of task difficulty and speed-accuracy instructions. Neuropsychologia *75*, 577-587.

S2. Zavala, B.A., Tan, H., Little, S., Ashkan, K., Hariz, M., Foltynie, T., Zrinzo, L., Zaghloul, K.A., and Brown, P. (2014). Midline frontal cortex low-frequency activity drives subthalamic nucleus oscillations during conflict. J Neurosci *34*, 7322-7333.

S3. Zavala, B.A., Tan, H., Little, S., Ashkan, K., Hariz, M., Foltynie, T., Zrinzo, L., Zaghloul, K.A., and Brown, P. (2015). Decisions made with less evidence involve higher levels of cortico-STN theta band synchrony. Journal of Cognitive Neuroscience *in revision*.

S4. Peirce, J.W. (2007). PsychoPy--Psychophysics software in Python. J Neurosci Methods *162*, 8-13.

S5. Cavanagh, J.F., Wiecki, T.V., Cohen, M.X., Figueroa, C.M., Samanta, J., Sherman, S.J., and Frank, M.J. (2011). Subthalamic nucleus stimulation reverses mediofrontal influence over decision threshold. Nat Neurosci *14*, 1462-1467.

S6. Zavala, B., Brittain, J.S., Jenkinson, N., Ashkan, K., Foltynie, T., Limousin, P., Zrinzo, L., Green, A.L., Aziz, T., Zaghloul, K., et al. (2013). Subthalamic nucleus local field potential activity during the Eriksen flanker task reveals a novel role for theta phase during conflict monitoring. J Neurosci *33*, 14758-14766.

S7. Hammond, C., Bergman, H., and Brown, P. (2007). Pathological synchronization in Parkinson's disease: networks, models and treatments. Trends Neurosci *30*, 357-364.

S8. Wiecki, T.V., Sofer, I., and Frank, M.J. (2013). HDDM: Hierarchical Bayesian estimation of the Drift-Diffusion Model in Python. Front Neuroinform *7*, 14.

S9. Frank, M.J., Gagne, C., Nyhus, E., Masters, S., Wiecki, T.V., Cavanagh, J.F., and Badre, D. (2015). fMRI and EEG predictors of dynamic decision parameters during human reinforcement learning. J Neurosci *35*, 485-494.

S10. Ratcliff, R., and McKoon, G. (2008). The diffusion decision model: theory and data for two-choice decision tasks. Neural Comput *20*, 873-922.

S11. Zhang, J., and Rowe, J.B. (2014). Dissociable mechanisms of speed-accuracy tradeoff during visual perceptual learning are revealed by a hierarchical drift-diffusion model. Front Neurosci *8*, 69.

S12. Bogacz, R., and Cohen, J.D. (2004). Parameterization of connectionist models. Behav Res Methods Instrum Comput *36*, 732-741.

S13. Frank, M.J., Samanta, J., Moustafa, A.A., and Sherman, S.J. (2007). Hold your horses: impulsivity, deep brain stimulation, and medication in parkinsonism. Science *318*, 1309-1312.

S14. Ridderinkhof, K.R., Forstmann, B.U., Wylie, S.A., Burle, B., and van den Wildenberg, W.P.M. (2011). Neurocognitive mechanisms of action control: resisting the call of the Sirens. . WIREs Cogn Sci *2*, 174–192.

S15. Wiecki, T.V., and Frank, M.J. (2013). A computational model of inhibitory control in frontal cortex and basal ganglia. Psychol Rev *120*, 329-355.

S16. Cohen, M.X. (2014). A neural microcircuit for cognitive conflict detection and signaling. Trends Neurosci *37*, 480-490.
