# Supplementary material for: Neural Correlates of Decision Thresholds in the Human Subthalamic Nucleus
Source: Curr Biol. 2016 Apr 4;26(7):916–20. doi: 10.1016/j.cub.2016.01.051 (PMC4826434; doi:10.1016/j.cub.2016.01.051)
Supplement: Document S2. Article plus Supplemental Information [file mmc2.pdf]

Current Biology

Report

Neural Correlates of Decision Thresholds in the Human Subthalamic Nucleus

Highlights

- Subjects make a choice when the integrated evidence crosses a "decision threshold"
- Such thresholds are reflected by 2- to 8-Hz oscillations in the subthalamic nucleus
- The relationship between decision thresholds and STN activity is context dependent
- This is mediated by altered prefrontal-STN coupling during high cautiousness

Authors

Damian M. Herz, Baltazar A. Zavala, Rafal Bogacz, Peter Brown

Correspondence

peter.brown@ndcn.ox.ac.uk

In Brief

In this article, Herz et al. show that decision thresholds in humans are modulated by subthalamic low-frequency oscillatory activity. This relationship depends on the subjects' level of cautiousness, which is mediated by increased influence of the medial prefrontal cortex-subthalamic pathway on decision thresholds when caution is required.

CrossMark

Herz et al., 2016, Current Biology *26*, 916–920
April 4, 2016 ©2016 The Authors
http://dx.doi.org/10.1016/j.cub.2016.01.051

CellPress

Cell Press

Current Biology
Report

Neural Correlates of Decision Thresholds in the Human Subthalamic Nucleus

Damian M. Herz,[1,2] Baltazar A. Zavala,[2,3] Rafal Bogacz,[1,2,4] and Peter Brown[1,2,4,*]
[1]Medical Research Council Brain Network Dynamics Unit at the University of Oxford, Mansfield Road, Oxford OX1 3TH, UK
[2]Nuffield Department of Clinical Neurosciences, University of Oxford, Level 6, West Wing, John Radcliffe Hospital, Oxford OX3 9DU, UK
[3]Surgical Neurology Branch, National Institutes of Health, 10 Center Drive, 3D 20, Bethesda, MD 20814, USA
[4]Co-senior author
*Correspondence: peter.brown@ndcn.ox.ac.uk
http://dx.doi.org/10.1016/j.cub.2016.01.051
This is an open access article under the CC BY license (http://creativecommons.org/licenses/by/4.0/).

SUMMARY

If humans are faced with difficult choices when making decisions, the ability to slow down responses becomes critical in order to avoid suboptimal choices. Current models of decision making assume that the subthalamic nucleus (STN) mediates this function by elevating decision thresholds, thereby requiring more evidence to be accumulated before responding [1–9]. However, direct electrophysiological evidence for the exact role of STN during adjustment of decision thresholds is lacking. Here, we show that trial-by-trial variations in STN low-frequency oscillatory activity predict adjustments of decision thresholds before subjects make a response. The relationship between STN activity and decision thresholds critically depends on the subjects' level of cautiousness. While increased oscillatory activity of the STN predicts elevated decision thresholds during high levels of cautiousness, it predicts decreased decision thresholds during low levels of cautiousness. This context-dependent relationship may be mediated by increased influence of the medial prefrontal cortex (mPFC)-STN pathway on decision thresholds during high cautiousness. Subjects who exhibit a stronger increase in phase alignment of low-frequency oscillatory activity in mPFC and STN before making a response have higher decision thresholds and commit fewer erroneous responses. Together, our results demonstrate that STN low-frequency oscillatory activity and corresponding mPFC-STN coupling are involved in determining how much evidence subjects accumulate before making a decision. This finding might explain why deep-brain stimulation of the STN can impair subjects' ability to slow down responses and can induce impulsive suboptimal decisions.

RESULTS AND DISCUSSION

The main goal of this study was to test whether neural activity of the subthalamic nucleus (STN) is related to modulations of decision thresholds during perceptual decision making. This has been suggested by computational models of decision making [1, 4] and studies using fMRI [3, 6]. Here, we directly recorded STN local field potentials (LFPs) in Parkinson's disease (PD) patients through electrodes implanted in the STN several days after deep-brain stimulation (DBS) surgery, while patients performed two versions of a moving dots task [10]. In both tasks, coherence rates of the moving dots linearly increased over time until 50% of all dots moved coherently in one direction. Participants pressed a button with their right or left index finger as soon as they perceived that the majority of dots were moving in the right or left direction. This design allowed us to assess neural activity, which is not related to abrupt stimulus changes or motor preparation, because changes in spectral STN activity were observed well before any choice was executed. Combining single-trial LFP analysis and drift diffusion modeling (DDM) allowed us to elucidate context-dependent relationships between single-trial oscillatory STN activity and features of decision making, which are not evident with conventional analyses of reaction times (RTs) and accuracy rates. For a detailed analysis of trial-averaged time frequency spectra related to the tasks, the reader is referred to previous reports by Zavala and colleagues [11, 12].

In task A, differences in the rate at which dots increased coherence were used to alter the rate of sensory evidence accumulation (left column in Figure 1A). Trials with low unidirectional coherence had significantly higher RTs relative to trials with medium unidirectional coherence (mean RT increase 38.1% ± 13.5 SD, $z_{(10)} = 2.934$, $P_{corrected} = 0.012$). Conversely, trials with high unidirectional coherence had significantly lower RTs relative to medium unidirectional coherence (mean RT decrease 22.4% ± 8.1 SD, $z_{(10)} = -2.934$, $P_{corrected} = 0.012$). Changing coherence in task A did not affect accuracy rates (change in accuracy during low unidirectional coherence relative to medium unidirectional coherence −3.7% ± 7.7 SD, $z_{(10)} = -1.481$, $P_{corrected} = 0.556$; change in accuracy during high unidirectional coherence relative to medium unidirectional coherence −1.8% ± 4.1 SD, $z_{(10)} = -1.680$, $P_{corrected} = 0.372$), see Figures 1B–1D. In task B, in 50% of trials the number of dots moving coherently *both* to the right and left increased until 0.83 s, after which the dots moving into the incorrect direction no longer increased in coherence, while the dots moving into the correct direction further increased coherence (right column in Figure 1A). There was thus no relative evidence for either direction in the first 0.83 s, particularly as neural integrators are thought to integrate the difference in dot coherence [13]. The remaining trials in task B

CellPress

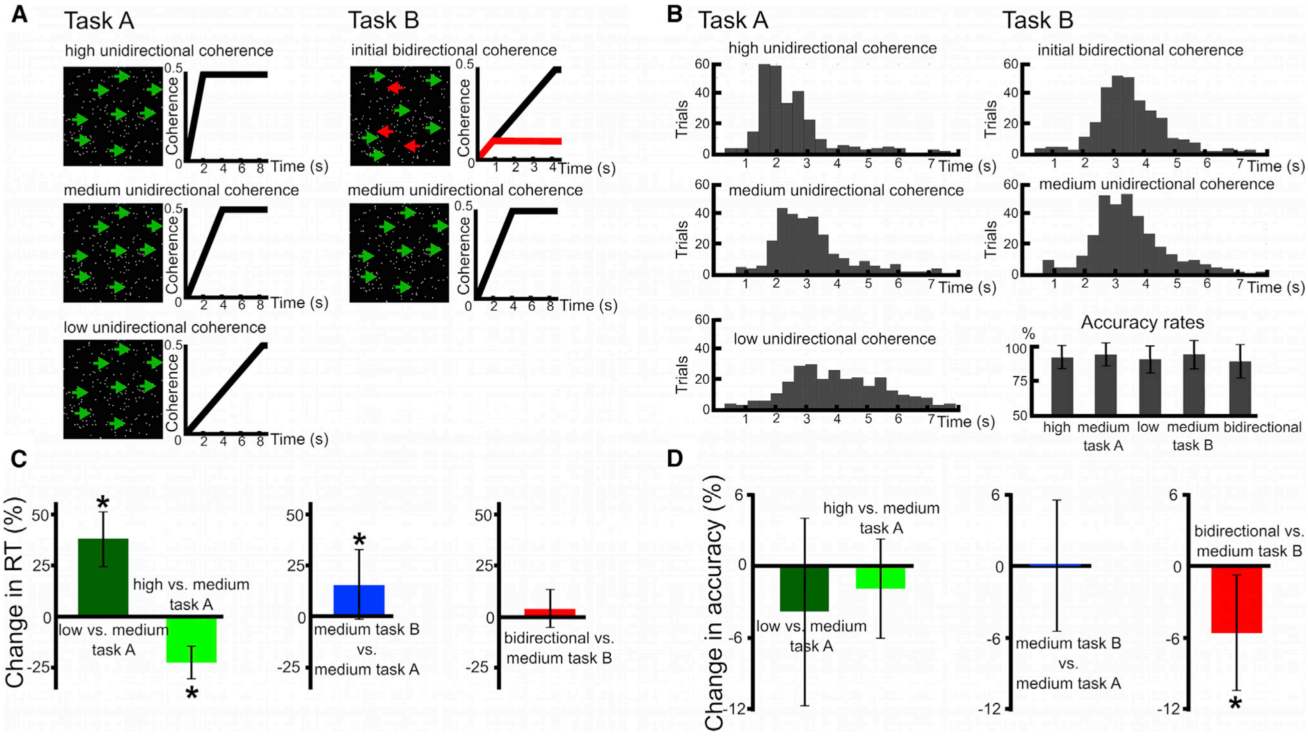

Figure 1. Experimental Tasks and Behavioral Analyses

(A) Experimental tasks A and B. In task A (first column), the rate of coherently moving dots changed between conditions (low, medium, and high unidirectional coherence). Black traces illustrate how coherence changed over time in the different conditions. In task B, 50% of trials showed dots moving coherently in opposite directions until dots moving in the incorrect direction were capped (red trace in right upper panel), while the remaining 50% of trials were identical to medium unidirectional coherence trials in task A.

(B) RT histograms and accuracy rates of all conditions are shown.

(C and D) Effects of the experimental manipulations on RT and accuracy. Columns reflect delta values. Error bars indicate SD. Asterisks indicate significance at $P_{corrected}$ 0.05.

were identical to trials with medium unidirectional coherence in task A. However, RTs in these trials in task B were significantly higher compared to identical trials in task A (relative increase in RT: 15.7% ± 17.1 SD, $z_{(10)}$ = 2.578, $P_{corrected}$ = 0.040), while accuracy was similar (change in accuracy 0.1% ± 5.6 SD, $z_{(10)} = -0.105$, $P_{uncorrected}$ = 0.917; Figures 1B–1D). This observation was in line with our a priori hypothesis that the presence of intermixed trials with initial bidirectional coherence in task B increased patients' level of cautiousness. Thus, they accumulated more evidence before making a decision in trials with medium unidirectional coherence in task B compared to task A. Finally, in task B RTs were similar in trials with initial bidirectional coherence and trials with unidirectional medium coherence (difference in RT: 4.3% ± 9.4% SD, $z_{(10)}$ = 1.511, $P_{corrected}$ = 0.524), while accuracy significantly decreased by 5.6% ± 4.9% SD ($z_{(10)} = -2.668$, $P_{corrected}$ = 0.032; Figures 1B–1D). This finding indicates that participants committed more erroneous responses in trials with initial bidirectional coherence when they did not accumulate sufficient evidence.

In order to test whether the observed behavioral effects could be related to modulation of the rate of evidence accumulation and decision thresholds, we modeled these latent processes underlying the observed behavior in the drift diffusion framework [14]. In DDM, sensory evidence is accumulated over time until the integrated evidence crosses the decision threshold and the choice is executed (see third column in Figure 2A). We applied a hierarchical Bayesian estimation of DDM parameters (HDDM), which is particularly suited for studies with relatively few trials [15]. As expected from the behavioral results, changing the amount of coherently moving dots significantly modulated drift rates; i.e., drift rates were lower in trials with low unidirectional coherence and initial bidirectional coherence and higher in trials with high unidirectional coherence compared to trials with medium unidirectional coherence (100% posterior probability for all effects being different than 0). Including trials with initial bidirectional coherence in task B significantly elevated decision thresholds, i.e., thresholds were higher in task B compared to task A (100% posterior probability). Please see Figure S1 and Supplemental Experimental Procedures for more details. This model had much stronger evidence compared to models proposing only changes in drift rate (difference in deviance information criterion [DIC], 34) or threshold (difference in DIC 121) and adequately predicted the observed behavior (Figure S1). We additionally validated HDDM by applying a non-hierarchical DDM (NHDDM) to the data, which yielded highly similar model parameter estimates at the group and individual subject level (Figure S2), and by applying HDDM to a simulated dataset (Figure S3; see also Supplemental Experimental Procedures). The

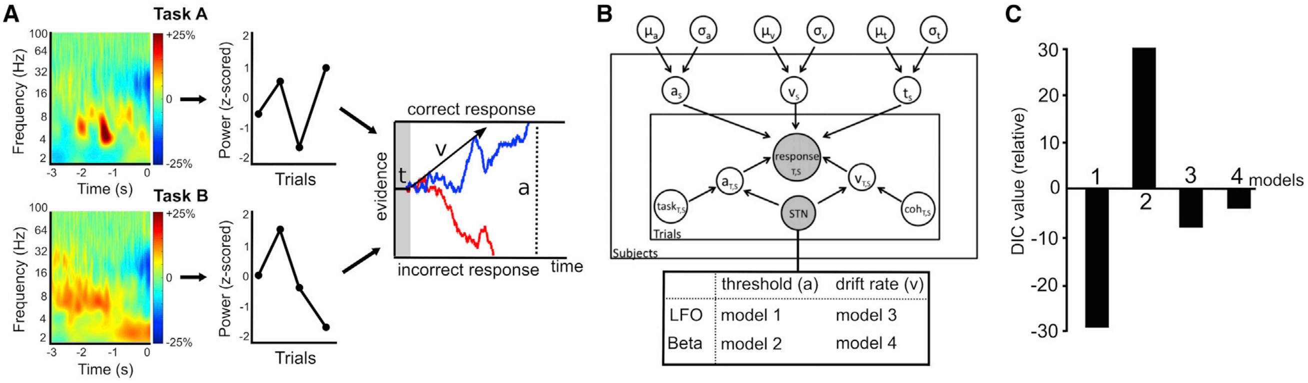

Figure 2. HDDM Analysis

(A) The time frequency plots show a pre-response increase in LFO power (time 0 indicates the response) relative to baseline averaged across conditions in both tasks (first column). Single trial LFPs were Z-scored for each task separately before entering them into the HDDM (second column). In DDM, *t* is the non-decision time (e.g., related to afferent delays and motor execution), and *v* is the drift rate indicating the rate of evidence accumulation until threshold *a* is reached and the response is executed (third column). Blue and red traces are examples of a single correct and incorrect response, respectively. Please note that this is a schematic illustration and does not show the actual model parameters.

(B) Illustration of HDDM. Parameters *a*, *v*, and *t* were estimated simultaneously for the group (circles outside the plates with group mean μ and variance σ) and subjects *S* (circles in outer plate). Variations in *a* and *v* were modulated by experimental manipulations (coh, coherence: trials with low and high unidirectional coherence and trials with initial bidirectional coherence relative to medium unidirectional coherence; task, task B relative to task A) at each trial *T* (circles in inner plate). Observed data are represented by shaded circles. They comprised responses (with RT and accuracy) and single-trial STN activity. The four neural HDDMs, which were compared, are shown in the box under the HDDM graphic. Please see Figure S1 for parameters of the HDDM without neural data and Figures S2 and S3 for validation of the HDDM.

(C) Model comparison. DIC values are shown relative to DIC of the HDDM not containing any neural data. Relative DIC were −29 (model 1), +30 (model 2), −8 (model 3), and −4 (model 4).

observation that participants did not have significantly longer RTs in trials with an initial bidirectional coherence compared to trials with medium unidirectional coherence in task B indicates that decision thresholds might have changed not only between tasks, but also between conditions in task B. Allowing thresholds to change between conditions in task B in HDDM showed that thresholds were higher in both conditions in task B compared to task A (> 99% posterior probability), but also higher in the medium unidirectional coherence trials in task B compared to trials with initial bidirectional coherence (>99% posterior probability). Nevertheless, as the main aim of this study was to investigate the role of the STN during perceptual decision making, we used a less complex, a-priori-defined model postulating thresholds adjustments between tasks, not conditions, below.

Accordingly, we assessed whether trial-by-trial measurements of STN activity—as reflected by LFP changes before the response—modulated different latent decision-making parameters at each trial using HDDM regression analysis. To this end, we computed single trial estimates of STN power in the time period preceding participants' responses (from −3 s until the response) and Z-scored these values separately for task A and B before including them in the HDDM (Figures 2A and 2B). We then estimated and compared four neural HDDMs based on a-priori-defined hypotheses, which differed in the precise frequency range of STN-LFP activity (2–8 Hz low-frequency oscillations [LFOs] versus 13–30 Hz beta oscillations) and the latent variable, which was modified by STN activity (threshold versus drift rate). Of note, there was no significant overall difference in pre-response STN LFO power between task A and B ($t = 1.646$, $p = 0.115$). Allowing trial-by-trial STN-LFO to modulate threshold estimates in the HDDM significantly improved model evidence compared to the model not containing any neural data (difference in DIC 29), and also clearly outperformed the alternative neural HDDMs (Figure 2C). Thus, model selection provided strong evidence that trial-by-trial variations in decision thresholds are modulated by STN-LFO.

Next, we aimed to investigate the exact relationship between STN-LFO and decision thresholds. To this end, we analyzed how decision thresholds varied as a function of STN LFO during both tasks by inspecting the posterior probability distribution of model parameters. We found a significant main effect of task, a significant main effect of STN-LFO and, critically, a significant interaction between task and STN-LFO (100% posterior probability for all parameters being different than 0, see Figure 3A). This interaction indicates that the effect of STN-LFO on decision thresholds critically depends on the level of cautiousness, which was higher in task B (see above). These results did not change when using non-Z-scored single trial estimates of STN activity or when using different wavelet lengths for computing STN power (see Supplemental Experimental Procedures). Post hoc tests of the effect of STN-LFO in task A (low cautiousness) and task B (high cautiousness) revealed that high power of STN-LFO predicted decreased decision thresholds in task A (100% posterior probability), while it predicted elevated decision thresholds in task B (95% posterior probability; Figure 3B). This context-dependent relationship did not change when using a more complex model where thresholds could vary between all conditions. In this additional control analysis all significant regression coefficients were negative in task A (100% probability for trials with low and high unidirectional coherence) and positive in task B (100% probability for trials with initial bidirectional coherence).

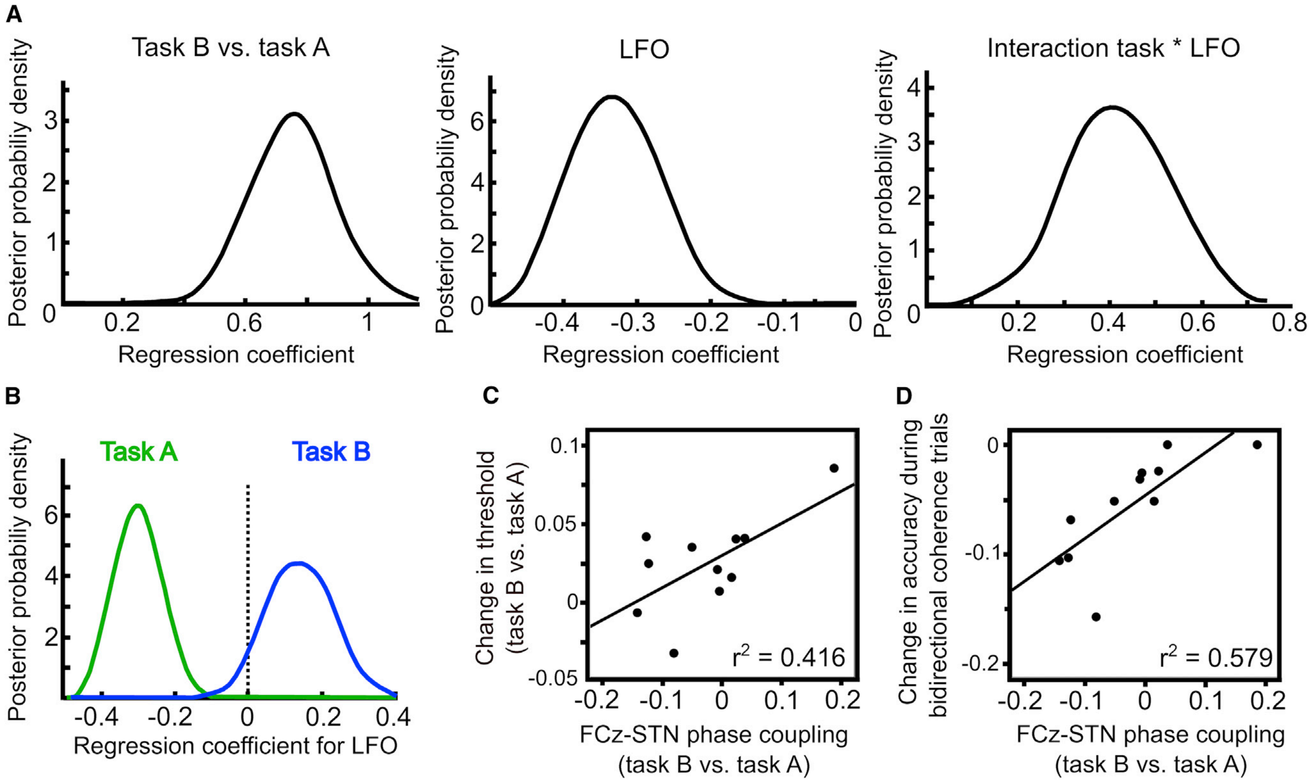

Figure 3. Neural Modulations of Decision Thresholds

(A) Posterior probabilities for modulation of decision thresholds by task (task B relative to task A), LFO, and their interaction. Peaks reflect the best estimates, while width represents uncertainty.

(B) Post hoc analysis showed an opposite relationship between LFO and thresholds for task A and B.

(C) Second (group) level regression between change in FCz-STN coupling (task B versus task A) and adjustments of decision thresholds derived from NHDDM ($p = 0.032$).

(D) Regression between change in FCz-STN coupling and participants' ability to control erroneous responses during trials with initial bidirectional coherence ($p = 0.007$).

These results indicate that STN activity, as reflected by LFO, does not simply reflect increases in decision thresholds, but that this relationship critically depends on the level of cautiousness. A possible explanation for this observation is a flexible reorganization of cortico-STN networks depending on task demands enabling the medial prefrontal cortex (mPFC) to increase its influence over STN function [2–4, 7, 9, 11, 16]. To test this hypothesis, we analyzed connectivity between electroencephalography (EEG) electrode FCz and STN by computing the intersite-phase clustering (IPC) (see Supplemental Experimental Procedures) reflecting how reliably the phases of oscillations in FCz and STN were aligned prior to the response. We then tested whether the extent to which IPC changed between task A and B predicted how much participants adjusted their decision thresholds estimated using NHDDM. This analysis showed that while there were no overall changes in FCz-STN IPC between tasks ($z_{(10)} = 1.067$, $p = 0.286$) the extent to which participants increased FCz-STN IPC significantly predicted adjustments in decision thresholds ($r^2 = 0.416$, $p = 0.032$), see Figure 3C. Furthermore, adjustments in FCz-STN IPC also predicted participants' ability to control erroneous responses ($r^2 = 0.579$, $p = 0.007$), see Figure 3D. Of note, these results stayed significant even when accounting for individual differences in drift rates (thresholds: $r^2 = 0.413$, $p = 0.045$; accuracy: $r^2 = 0.601$, $p = 0.008$). These results suggest that mPFC-STN communication through phase alignment might be an important mechanism for adjusting decision thresholds and thereby controlling erroneous responses when participants are more careful in making decisions, although it should be noted that regression analyses were based on relatively few observations ($n = 11$).

In conclusion, we report three novel findings in this study. First, our results demonstrate for the first time that oscillatory STN activity reflects trial-by-trial modulations of decision thresholds, i.e., how much evidence subjects integrate before making a decision. This relationship is specific for the latent mechanism underlying decision making (thresholds, but not drift rates) and frequency range of oscillatory activity (LFO, but not beta oscillations). Second, we show that STN activity does not uni-directionally increase decision thresholds but can have opposing effects on thresholds depending on subjects' level of cautiousness. Finally, we found that modulations of the phase alignment between mPFC and STN, a mechanism that might optimize information transfer between these two regions [17], predicts adjustments of decision thresholds and participants ability to control erroneous responses. Thus, a context-dependent integration of STN in dynamic cortico-STN networks might be critical in the

ability to adjust behavior to changing environments and give rise to the context-specific relationships between STN activity and modulation of decision thresholds observed in this study. This neural mechanism might be affected in individuals who express impulsive behavior during therapeutic stimulation of the STN [2, 4, 5]. It remains to be elucidated whether such unwanted effects of DBS can be avoided by specifically targeting abnormal (beta) oscillations in PD [18] leaving modulations of LFO relatively intact.

SUPPLEMENTAL INFORMATION

Supplemental Information includes three figures, one table, and Supplemental Experimental Procedures and can be found with this article online at http://dx.doi.org/10.1016/j.cub.2016.01.051.

AUTHOR CONTRIBUTIONS

B.A.Z. and P.B. designed the experiments. D.M.H. and R.B. conducted the drift diffusion modeling analysis. D.M.H. and B.A.Z. conducted the electrophysiological analysis. P.B. supervised the project. D.M.H. wrote the first draft of the manuscript. B.A.Z., R.B., and P.B. contributed to editing and revising the manuscript.

ACKNOWLEDGMENTS

We would like to thank H. Tan, S. Little, K. Ashkan, M. Hariz, T. Foltynie, L. Zrinzo, and K. Zaghloul for their contributions to acquisition and analysis of the primary data and the patients for volunteering to take part in the study. All patients provided written informed consent to participate, in accordance with the Declaration of Helsinki, and the study was approved by the local ethics committee. This project has received funding from the Medical Research Council (award MC_UU_12024/1) and the European Union's Horizon 2020 research and innovation program under Marie Sklodowska-Curie grant agreement 655605. Original behavioral and electrophysiological data are available on request.

Received: November 13, 2015
Revised: January 11, 2016
Accepted: January 22, 2016
Published: March 17, 2016

REFERENCES

1. Bogacz, R., and Gurney, K. (2007). The basal ganglia and cortex implement optimal decision making between alternative actions. Neural Comput. *19*, 442–477.
2. Cavanagh, J.F., Wiecki, T.V., Cohen, M.X., Figueroa, C.M., Samanta, J., Sherman, S.J., and Frank, M.J. (2011). Subthalamic nucleus stimulation reverses mediofrontal influence over decision threshold. Nat. Neurosci. *14*, 1462–1467.
3. Frank, M.J., Gagne, C., Nyhus, E., Masters, S., Wiecki, T.V., Cavanagh, J.F., and Badre, D. (2015). fMRI and EEG predictors of dynamic decision parameters during human reinforcement learning. J. Neurosci. *35*, 485–494.
4. Frank, M.J., Samanta, J., Moustafa, A.A., and Sherman, S.J. (2007). Hold your horses: impulsivity, deep brain stimulation, and medication in parkinsonism. Science *318*, 1309–1312.
5. Green, N., Bogacz, R., Huebl, J., Beyer, A.K., Kühn, A.A., and Heekeren, H.R. (2013). Reduction of influence of task difficulty on perceptual decision making by STN deep brain stimulation. Curr. Biol. *23*, 1681–1684.
6. Mansfield, E.L., Karayanidis, F., Jamadar, S., Heathcote, A., and Forstmann, B.U. (2011). Adjustments of response threshold during task switching: a model-based functional magnetic resonance imaging study. J. Neurosci. *31*, 14688–14692.
7. Richard Ridderinkhof, K., Forstmann, B.U., Wylie, S.A., Burle, B., and van den Wildenberg, W.P.M. (2011). Neurocognitive mechanisms of action control: resisting the call of the Sirens. Wiley Interdiscip. Rev. Cogn. Sci. *2*, 174–192.
8. Wei, W., Rubin, J.E., and Wang, X.J. (2015). Role of the indirect pathway of the basal ganglia in perceptual decision making. J. Neurosci. *35*, 4052–4064.
9. Wiecki, T.V., and Frank, M.J. (2013). A computational model of inhibitory control in frontal cortex and basal ganglia. Psychol. Rev. *120*, 329–355.
10. Gold, J.I., and Shadlen, M.N. (2007). The neural basis of decision making. Annu. Rev. Neurosci. *30*, 535–574.
11. Zavala, B.A., Tan, H., Little, S., Ashkan, K., Hariz, M., Foltynie, T., Zrinzo, L., Zaghloul, K.A., and Brown, P. (2014). Midline frontal cortex low-frequency activity drives subthalamic nucleus oscillations during conflict. J. Neurosci. *34*, 7322–7333.
12. Zavala, B., Tan, H., Little, S., Ashkan, K., Green, A.L., Aziz, T., Foltynie, T., Zrinzo, L., Zaghloul, K., and Brown, P. (2016). Decisions made with less evidence involve higher levels of cortico-STN theta band synchrony. J. Cogn. Neurosci. Published online February 4, 2016. http://dx.doi.org/10.1162/jocn_a_00934.
13. Mazurek, M.E., Roitman, J.D., Ditterich, J., and Shadlen, M.N. (2003). A role for neural integrators in perceptual decision making. Cereb. Cortex *13*, 1257–1269.
14. Ratcliff, R., and McKoon, G. (2008). The diffusion decision model: theory and data for two-choice decision tasks. Neural Comput. *20*, 873–922.
15. Wiecki, T.V., Sofer, I., and Frank, M.J. (2013). HDDM: Hierarchical Bayesian estimation of the Drift-Diffusion Model in Python. Front. Neuroinform. *7*, 14.
16. Cohen, M.X. (2014). A neural microcircuit for cognitive conflict detection and signaling. Trends Neurosci. *37*, 480–490.
17. Fries, P. (2015). Rhythms for cognition: communication through coherence. Neuron *88*, 220–235.
18. Little, S., Pogosyan, A., Neal, S., Zavala, B., Zrinzo, L., Hariz, M., Foltynie, T., Limousin, P., Ashkan, K., FitzGerald, J., et al. (2013). Adaptive deep brain stimulation in advanced Parkinson disease. Ann. Neurol. *74*, 449–457.

Current Biology, Volume *26*

Supplemental Information

Neural Correlates of Decision Thresholds in the Human Subthalamic Nucleus

Damian M. Herz, Baltazar A. Zavala, Rafal Bogacz, and Peter Brown

Supplemental information

Supplemental figures:

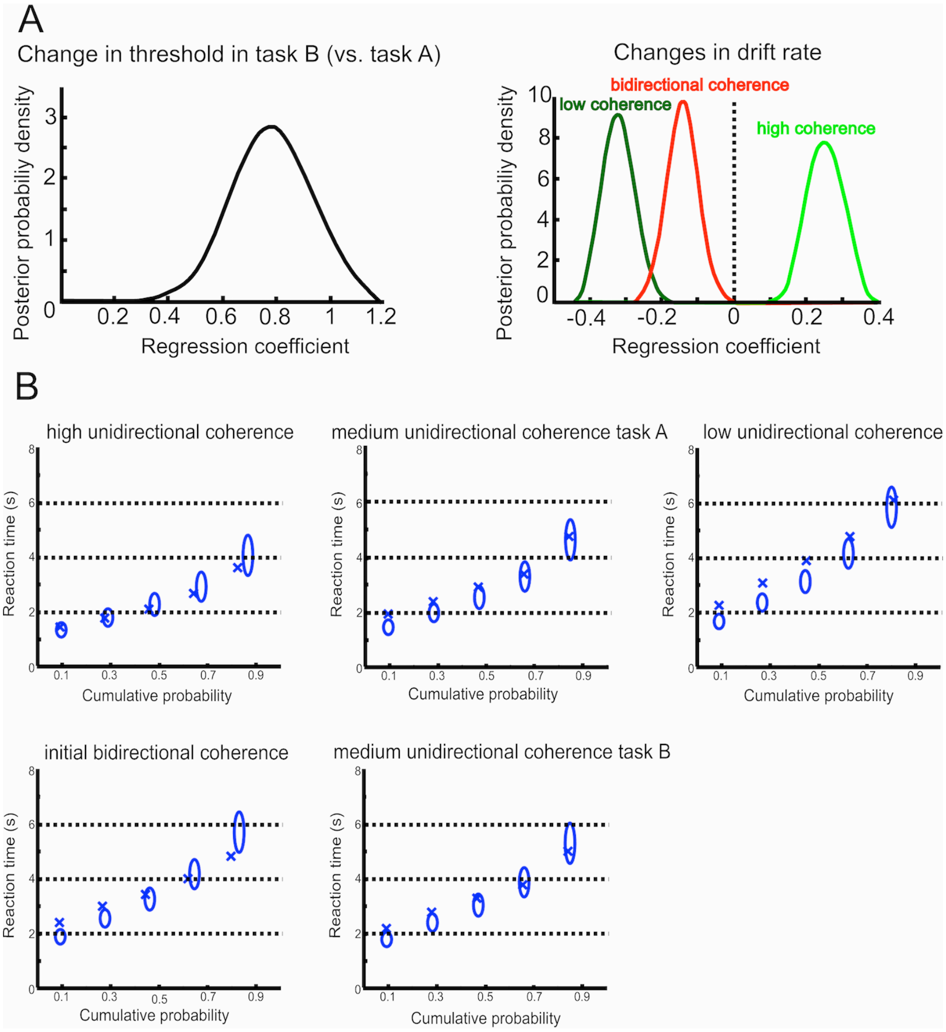

Figure S1: HDDM without neural data. Related to figure 2. (A) Main effects on decision thresholds and drift rates of the HDDM not containing any neural data. The left panel shows the posterior distribution of the regression coefficient for the effects on the decision thresholds. The right panel shows the posterior distribution of coefficients for the effects on drift rates. Peaks reflect the best parameter estimate, while the width of the distribution represents uncertainty. All posterior distributions are shifted away from zero (i.e. they do not overlap with 0 on the x-axis) and thus have 100% posterior probability to be different than 0. Note that threshold regression coefficients (left panel) are shown relative to task A, i.e. the threshold estimate during task A constitutes the intersect in the regression. Similarly, drift rate regression coefficients (right panel) are shown relative to drift rate during medium coherence (see also Supplemental Experimental Procedures). (B) Quantile probability plots. Observed mean reaction times (RT) for five quantiles (10, 30, 50, 70 and 90 %) are marked by a cross and plotted against their cumulative probability for the five conditions separately. Predicted quantile mean RTs are marked as ellipses with the width capturing estimation uncertainty (standard deviation of the posterior predictive distribution from the model). The plot shows that the HDDM provided overall good predictions of the observed data and, more importantly, captured effects of task manipulations on RT. For example, it predicts an RT shift upwards from high to low unidirectional coherence trials as well as increased RT in medium unidirectional coherence trials in task B compared to medium unidirectional coherence trials in task A. Due to the low number of error trials quantile probability plots are only shown for correct trials. Nevertheless, predicted error rates can be evaluated by the predicted cumulative probabilities along the x-axis (e.g. poor predictions of error rates would results in incongruencies between predicted and observed quantile RT along the x-axis).

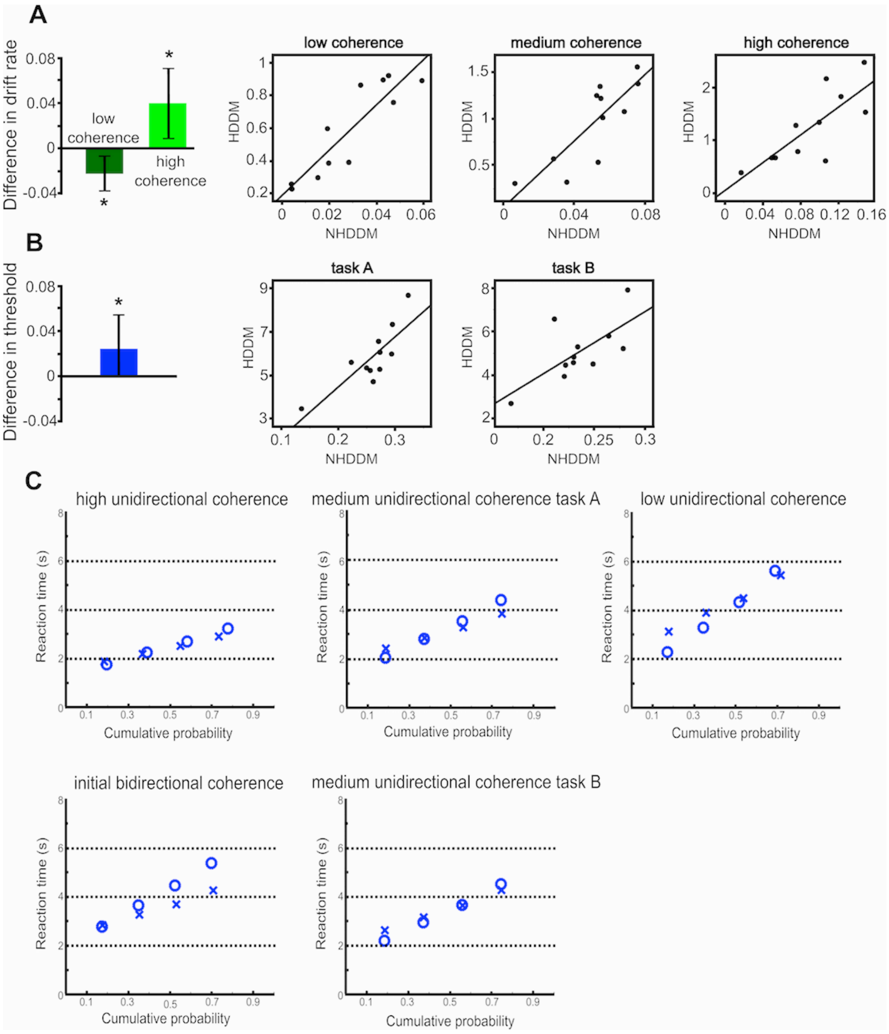

Figure S2: Non-hierarchical DDM (NHDDM). Related to figure 2. (A) Drift rate slope parameters estimated from the NHDDM showed a significant difference in drift rate slopes in low compared to medium unidirectional coherence trials (-0.022 ± 0.015 SD, $z_{(10)}$ = -2.756, $P_{corrected}$ = 0.012) and high compared to medium unidirectional coherence trials (0.040 ± 0.031 SD, $z_{(10)}$ = 2.845, $P_{corrected}$ = 0.008; Wilcoxon signed rank tests). Thus, the mean effects of coherence manipulations on drift rates were similar to the ones obtained from the HDDM (figure S1). Note that in the NHDDM there was no separate drift rate slope for trials with initial bidirectional coherence, since these drift rate slopes were modeled as medium drift rate slopes with an 0.83 s delay according to the delayed onset of relative increase in evidence accumulation in this condition. The correlation plots show that drift rate parameters estimated from NHDDM and HDDM were also proportionally similar, i.e. participants with high drift rate slopes in NHDDM had high drift rates in the HDDM and vice versa (low coherence: rho 0.892, $P_{corrected}$ = 0.003, medium coherence: rho 0.824, $P_{corrected}$ = 0.006, high coherence: rho 0.785, $P_{corrected}$ = 0.012; Pearson correlations). (B) Like the HDDM, NHDDM threshold parameters showed a significant increase in threshold in task B compared to task A (0.024 ± 0.031 SD, $z_{(10)}$ = 2.223, P = 0.026; Wilcoxon signed rank test). Furthermore, threshold parameters were also proportionally similar as indicated by correlation analyses (task A: rho 0.822, $P_{corrected}$ = 0.004, task B: rho 0.682, $P_{corrected}$ = 0.042, Pearson correlations). Note that parameters estimated in HDDM are scaled with a factor of 10 compared to 'traditional' DDM values (e.g. a drift rate of 0.1 in the NHDDM corresponds to a drift rate of 1 in HDDM). (C) Quantile probability plots. Observed mean RTs for four quantiles (20, 40, 60 and 80 %) are marked by a cross and plotted against their cumulative probability for the five conditions separately. Predicted quantile mean RTs are marked as circles. The plot shows that the NHDDM, like the HDDM, provided overall good predictions of the observed data and captured effects of task manipulations on RT (see also figure S1).

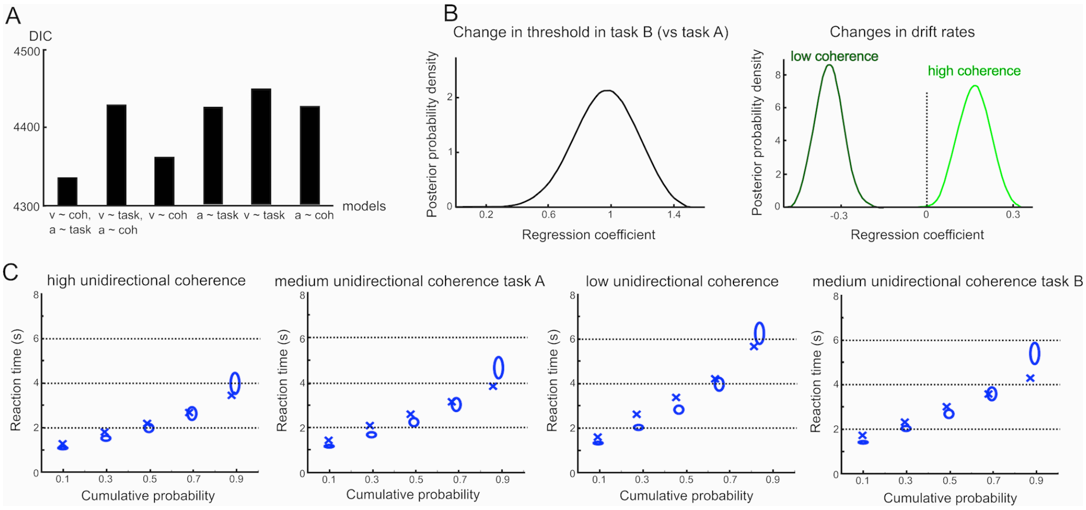

Figure S3: Results of the simulation analysis. Related to figure 2. A dataset (RT and accuracy) was simulated based on threshold and drift rate slope parameters of a representative patient (patient with parameters closest to the group mean). HDDM was the fitted to this simulated dataset. (A) We compared different models, which differed in the decisiosn parameter, which was modulated during the tasks (drift rate and threshold, only drift rate or only threshold) and the experimental manipulation, which modulated these latent parameters (coherence and task, only coherence and only task). The true model, which was used for simulating the dataset, consisted of a drift rate slope modulation by coherence rate and a threshold modulation by task. This model was also clearly the best model according to its DIC (first column in (A); DIC difference to next best model 27), i.e. HDDM model evidence favored the true model over the alternative models. DIC values for all models were: model S1 (a ~ task, v ~ coh) 4336, model S2 (v ~ task, a ~ coh) 4429, model S3 (v ~ coh) 4363, model S4 (a ~ task) 4426, model S5 (v ~ task) 4449, model S6 (a ~ coh) 4427. The recovered parameters are shown in (B), with a significant change in threshold in task B vs. task A and significant changes in drift rates in trials with low and high unidirectional coherence compared to trials with medium unidirectional coherence (100% posterior probability for all parameter estimates being different than 0). Interestingly, the model was also able to recover that the true drift rate manipulation during low unidirectional coherence was stronger than the change in drift rate during high unidirectional coherence (relative change in the drift rate slope used for simulation was -0.028 for low unidirectional coherence and +0.021 for high unidirectional coherence). (C) shows quantile probability plots with observed (cross) and predicted (ellipses) RT distribution for the four considered conditions. As in figure S1, the width of ellipses indicates estimation uncertainty (standard deviation).

Supplemental tables:

Patient	Age	Disease duration	UPDRS-III OFF	UPDRS-III ON	First symptom	Reason for surgery	Medication
1	49	10	42	6	Tremor	Tremor	Levodopa 300; Trihexyphenidyl 2
2	50	4	N/A	N/A	Tremor	Tremor	Levodopa 400; Rotigotine 16; Entacapone 600
3	66	16	32	13	Loss of dexterity	Bradykinesia	Levodopa 600; Ropinirole 24; Rasagiline 1; Amantadine 200;
4	51	7	58	13	Loss of sense of smell	Tremor, gait difficulties	Levodopa 1300
5	64	12	70	20	Tremor	Dyskinesias	Levodopa 1200; Apomorphine 7 mg/h
6	47	14	34	11	Bradykinesia	Dyskinesias, motor fluctuations	Levodopa 350; Pramipexole 1.05; Amantadine 300
7	66	14	63	24	Shoulder pain, stiffness	Motor fluctuations	Levodopa 650; Pergolide 9
8	57	6	21	7	Bradykinesia	Dyskinesias, motor fluctuations	Levodopa 750; Entacapone 1000
9	61	4	37	15	Tremor	Tremor	Levodopa 750; Amantadine 200; Entacapone 1000
10	65	15	51	21	Tremor	Freezing	Levodopa 400; Ropinirole 12; Amantadine 200
11	42	9	60	42	Loss of dexterity	Bradykinesia, dystonia, freezing	Levodopa 600; Amantadine 400
mean	56.2	10.1	46.8	17.2			

Table S1: Clinical and demographic characteristics of included patients. Age is given in years, medication is given in mg/day unless otherwise stated. N/A, not available.

Supplemental Experimental Procedures:

Participants

Eleven patients (six males, mean age 56.2 years ± 8.7 (standard deviation, SD), mean disease duration 10.1 years ± 4.4 SD) with Parkinson's disease (PD) undergoing deep brain stimulation (DBS) of the subthalamic nucleus (STN) were enrolled in the study (for clinical details see table S1). In all patients a quadripolar macroelectrode (model 3389, Medtronic Neurologic Division, Minneapolis, MN, USA) featuring four platinum-iridium cylindrical surfaces was implanted in bilateral STN. Lead localization was confirmed either by intra-operative stereotactic magnetic resonance imaging or by the clinical effect during the operation and immediate post-operative stereotactic computerized tomography. DBS electrode extension cables were externalized enabling recordings prior to implantation of a subcutaneous pacemaker up to seven days later.

A healthy control group could not be included in the study due to the invasive nature of electrode implementation. However, in order to approximate physiological function of STN as closely as possible, all recordings were conducted in patients on their normal medication. Importantly, the dopaminergic state in PD does not affect decision thresholds [S1], which were the main focus of this study. Mean improvement in motor function as indexed by Unified Parkinson's Disease Rating Scale-III was 63.9 % ± 14.8 SD after a levodopa challenge indicating a very good dopamine response in the studied patient group. All patients completed the two experimental tasks consecutively on the same day, three to six days after electrode implantation. In accordance with the Declaration of Helsinki, all

patients gave their written informed consent to participate in the study, which was approved by the local ethics committee. Of note, data of the same patient group has been reported previously concerning separate group analysis of averaged time frequency spectra during the tasks [S2, S3]. Here, for the first time, we demonstrate that trial-by-trial variations of STN activity (which are considered noise in averaged time-frequency spectra) predict context-dependent adaptations of decision-thresholds during perceptual decision-making.

Paradigms

The experimental tasks have been described in detail previously [S2, S3] and are illustrated in Fig. 1A. A cloud of 200 randomly moving white dots was presented on a black background. The tasks were presented on a 33 cm Macintosh laptop with a 60 Hz screen refresh rate using PsychoPy [S4]. The cloud was 14 cm in diameter and dots were 10 pixels large (visual angle ≈ 0.25°). Each randomly moving dot moved in a straight line at a rate of 0.14 mm per frame for 20 frames (333 ms) before moving to another part of the cloud and moving in a new direction chosen pseudorandomly between -180° and 180°. Participants were instructed to indicate the overall direction of dots whenever they noticed that the cloud was moving to the left or to the right side. They were instructed to respond as fast and accurately as possible, i.e. to balance speed and accuracy. Choices were indicated by a left ("z") or right ("/") button press on a keyboard with the left and right index finger, respectively. Participants performed task A first. The task comprised three randomly interspersed conditions. During trials with high unidirectional coherence the number of dots moving coherently into either the left or right side of the screen increased linearly from 0% to 50% within 2.083 s (corresponding to 0.004% per frame). During trials with medium and low unidirectional coherence 50% coherence was reached after 4.17 s (0.002% increase per frame) and 8.333 (0.001% increase per frame), respectively. In task B, participants were told that in some of the trials dots would start moving in opposite directions instead of just one direction. In these trials the number of dots moving coherently both to the right and left increased at the same rate until 0.83 s. After 0.83 s, the dots moving in the incorrect direction no longer increased in coherence (i.e. they were capped at 10% of all 200 dots), while the dots moving into the correct direction further increased their coherent motion until reaching 50% coherence after 4.17s. The remaining trials of task B were identical to trials with medium unidirectional coherence in task A (i.e. the number of dots moving coherently in one direction increased linearly from 0% to 50% within 4.17s). In both tasks, all trials were pseudo-randomly presented with equal probability (28 of 84 trials per condition in task A, 40 of 80 trials per condition in task B). Visual feedback ("incorrect") was provided for a duration of 0.75 s only in case of erroneous responses or prolonged response times > 14 s. Before each trial, dots moved randomly for a time period between 2 and 4 s. Prior to commencement of recordings participants could practice the tasks as long as they wished (usually < 10 trials).

We chose to use coherence rates, which linearly increased over time in contrast to time-invariant coherence differences between conditions in order to avoid presenting subjects with an explicit cue in the beginning of each trial. Thus, at no point subjects were given a 'cue' indicating that the trial had begun. After the subject made the response for a given trial, all dots immediately began to move in random direction for a duration of 2 - 4 s before slowing starting to move coherently again for the next trial. This design allowed us to assess neural activity, which is not related to abrupt stimulus changes or motor preparation, because the changes in spectral STN activity were observed well before the choice was executed [S2, S3].

Analysis of behavioral data

All trials with RT > 8 s or < 0.25 s were discarded from further analyses. There were three main experimental manipulations during the tasks. First, an increase (high unidirectional coherence) or decrease (low unidirectional coherence) in the rate of coherently moving dots relative to trials with medium unidirectional coherence in task A. Second, the difference between identical trials with medium unidirectional coherence rates in task B and task A, which only differed regarding the presence of intermixed trials with initial bidirectional coherence in task B. We hypothesized that the presence of such trials would increase participants' level of cautiousness due to the increased task demands. Third, the difference between trials with initial bidirectional coherence and medium unidirectional coherence trials in task B. The first 0.83s in the former trials did not convey any relative evidence for either direction.

To assess effects on reaction times (RT) and accuracy rates we computed the relative change in RT (e.g. (RT during high unidirectional coherence – RT during medium unidirectional coherence) / RT during medium unidirectional coherence) and accuracy rates (e.g. (Accuracy during high unidirectional coherence – Accuracy during medium unidirectional coherence) / Accuracy during medium unidirectional coherence) and used non-parametric one-sample Wilcoxon signed-rank tests to test the

resulting values against a median of 0 using SPSS statistics (v22, IBM, New York, USA). The significance threshold was set to 0.05 adjusting for multiple comparisons using the Bonferroni method.

Analysis of trial-by-trial LFPs

Local field potentials (LFP) were recorded from DBS electrodes. In addition, electroencephalography (EEG) was recorded from FCz, Cz and Pz according to the international 10-20 system. A wider coverage with EEG electrodes was not possible due to surgical wounds and dressings. Preprocessing of electrophysiological data was identical to the procedures reported previously [S2, S3]. In short, all signals were sampled at 2048 Hz, band-pass filtered between 0.5 and 500 Hz, amplified (TMSi porti, TMS International, Enschede, The Netherlands), down-sampled to 1000 Hz and notch filtered at 50 Hz. LFPs were then converted to a bipolar montage between contacts (three channels per STN) and EEG electrodes were re-referenced to Cz to limit effects of volume conduction. Trials with clear artifacts were discarded. After removal of trials with artifacts and behavioral outliers, 29.5 trials per subject and condition and 1621 trials in total remained. Power and phase of LFPs were computed using the continuous wavelet transform (1 Hz frequency resolution, 10 cycles per frequency) and resulting time frequency spectra were chunked into individual trials based on the onset of the motor responses for each task separately. To control that the relatively long wavelets did not lead to smearing of LFP power across trials, we repeated this analysis using 5 cycle wavelets. Single trial power estimates of low frequency oscillations (LFO, see below) using 10 cycle wavelets and 5 cycle wavelets, respectively, were highly correlated (rho = 0.95, Spearman correlation over all trials) and yielded identical results in the HDDM analysis (see below; data is only shown for the analysis using 10 cycle wavelets). Since group analysis of time-frequency spectra during the tasks showed a sustained pre-response increase in LFO from approximately 3 s prior to the button presses [S2, S3], see also fig. 2A, power values were extracted from -3 to 0 seconds. In case response time in a given trial was < 3 s (median RT was 3.113 s) time windows for extracting single-trial pre-response power values were restricted to –RT to 0 in order to avoid modeling STN activity prior to trial onset. Single trial estimates were then averaged across the respective time window and frequencies (2-8 Hz and 13-30 Hz, see below) and normalized to the mean power of those frequencies in the peri-response window (-3 to +2 s) of each participant and finally averaged across all STN electrodes. We chose to average across STN electrodes in order to avoid selection bias, even though this procedure might underestimate spectral changes. Importantly, neither STN -power, nor -FCz connectivity values differed regarding localization on the dorso-ventral STN axis [S2]. The 2-8 Hz window was chosen, because increases in LFO and cortico-STN connectivity in the current tasks did not have a clear lower boundary at 4 Hz (theta) [S2, S3], which is in agreement with previously reported motor conflict-related power changes in STN [S5, S6]. Beta power (13-30 Hz) was included, because of its central role in motor processing in the STN [S7]. To assess whether there were overall differences in pre-response LFO power between task A and B we compared power values (averaged over the 2-8 Hz frequency window and fixed -3 to 0s time window) using paired samples t-test.

Drift diffusion modeling

The goal of drift diffusion modeling (DDM) in this study, was to investigate whether trial-by-trial fluctuations in pre-response STN power were related to modulations of latent processes underlying perceptual decision making and whether this relationship was specific to the frequency range of oscillatory activity (LFO vs. Beta) and decision-making process (decision threshold vs. drift rate). A Bayesian hierarchical DDM (HDDM) has been developed and optimized for such purposes allowing regression of trial-by-trial variations in brain activity on decision parameters [S8]. Importantly this toolbox is also particularly suited for studies with low trial counts [S5, S8, S9], such as the current study with restrictions on the possible duration of the experiment due to vulnerability of the studied patient group. In DDM choices between two alternative options (here pressing the left or right button) are simulated by a noisy process in which evidence is accumulated over time (here the fraction of dots moving into different directions) until the evidence for one choice over the other is sufficient and the response is executed. There are two main parameters of interest in DDM. First, the rate of evidence accumulation is reflected by the drift rate v, which critically depends on the presented stimulus. Thus, in the current tasks drift rate was assumed to be modulated by the rate of change in coherently moving dots (manipulations in task A) and a delay in relative evidence accumulation due to dots moving into opposite directions (manipulation in task B). Second, the decision threshold a determines how much evidence has to be accumulated until a decision is made and thus reflects a measure of cautiousness. In this study, this parameter was assumed to be modulated by the presence of intermixed trials with an initial bidirectional coherence in task B, i.e. task B vs. task A (see also results of the behavioral analysis). A third parameter is the non-decision time t reflecting processes unrelated to the decision

(e.g. sensory processing in visual areas and motor execution). These parameters are estimated by the model based on the observed behavior, i.e. accuracy and response times. DDM has been shown to reliably detect these latent decision-making parameters throughout a variety of different behavioral and neurophysiological experiments over the last few decades [S10]. In the recently developed HDDM the trade-off between random and fixed effects models is optimized by assuming that parameters from individual subjects are drawn from the group distribution while allowing variation from this distribution given sufficient evidence to overwhelm the group prior [S8]. Parameters for each subject and condition were modelled according to a normal (real valued parameters) or Gamma distribution (positive valued parameters) centered on the group mean with group variance. Prior distributions were informed by 23 previous studies reporting parameters on a range of decision-making tasks [S8]. The starting parameter z (often referred to as bias parameter) was fixed to 0.5, because dots moving to the right or left had equal probability, i.e. participants were not biased toward right or left responses in the experimental tasks. Markov chain Monte Carlo sampling methods were used for accurate Bayesian approximation of the posterior distribution of parameters (generating 20 000 samples, discarding 10 000 samples as burn-in and keeping every fifth sample). We inspected traces of model parameters, their autocorrelation and computed the R-hat (Gelman-Rubin) convergence statistics to ensure that the models had properly converged [S8].

First we assessed basic assumptions of the model without including any neural data. This comprised modulations of drift rate by differences in the rate of coherence, where we a-priori expected a decrease in drift rate in trials with low unidirectional coherence and trials with an initial bidirectional coherence and an increase in drift rate in trials with high unidirectional coherence relative to trials with medium unidirectional coherence as well as modulations of threshold by task with an increased threshold in task B relative to task A. We validated our model assumptions (both drift rate *and* threshold were modulated by the experimental manipulations) by testing that this model had stronger evidence than models postulating only a change in drift rate or threshold respectively, using the deviance information criterion (DIC). DIC is widely used for comparisons of hierarchical models where other measures (e.g. Bayesian information criterion) are not appropriate [S8, S9]. A lower DIC value for a given model (for the whole group) indicates higher likelihood for that model compared to an alternative model taking into account model complexity (degrees of freedom). Usually a DIC difference of 10 is considered significant [S11]. Parameters of the best model were analyzed by Bayesian hypothesis testing, i.e. the percentage of samples drawn from the posterior that fall within a certain region (e.g. > 0). Posterior probabilities ≥ 95% were considered significant. Please note that this value is not equivalent to p-values estimated by frequentist methods (e.g. Wilcoxon signed rank tests during the analysis of behavioral data), but it can be interpreted in a similar manner. In the article, we refer to these estimates as *posterior probabilities* to clearly demarcate them from p-values. To assess model predictions we computed quantile probability plots, a popular measure for assessing model performance in the DDM framework [S10]. Here, observed and predicted RT for the 10, 30, 50, 70 and 90 percentile of trials (e.g. 10% fastest trials, 30% fastest trials, etc.) were plotted against their observed and predicted cumulative probability for each condition. Due to the low number of error trials, we only plotted quantiles for correct trials (see figure S1).

Of note, we made two simplifying assumptions when using HDDM. First, we did not explicitly model that a relative difference in coherence (i.e. to the left or right side) first started after 0.83 s in 50% of trials in task B, but just assumed a different drift rate during these trials. Furthermore, we assumed that changing the rate of dot coherence over time would have a similar effect on evidence accumulation as a difference in dots coherence, which is constant over time. This assumption was supported by the observation that participants executed responses during equal levels of integrated evidence and not instantaneous evidence in task A [S3]. To validate this approach we also applied a non-hierarchical DDM using custom-written scripts in matlab (R2015a, The MathWorks, Natick, MA, USA), in which we specified the exact properties of the task. In particular this model assumed that drift rate in condition i was a linearly increasing function $v_i(t)=s_i t$, where s_i is the slope parameter for condition i, and t is the time from onset of coherent motion, or the time from which coherence of dots moving in the correct direction was higher than the coherence of dots moving in the other direction on trials with an initial bidirectional coherence. We applied a previously validated method for non-hierarchical estimation of model parameters using a subplex algorithm for minimizing the cost function defined as the difference between observed and predicted quantile RT distribution (20, 40, 60 and 80 percentile) according to a least-squares estimation [S12]. The parameters comprised drift rate slopes s_1 (trials with low unidirectional coherence in task A), s_2 (trials with medium unidirectional coherence in task A and B including a delayed onset of 0.83 s for trials with an initial bidirectional coherence in task B) and s_3 (trials with high unidirectional coherence in task A), threshold a_1 (task A) and a_2 (task B) as well as the non-decision time t. The parameters were fitted to the observed data (10 iterations of

random search for starting values, 70 optimization iterations, 50 tuning iterations and 20 repetitions of the whole process; please see Bogacz and Cohen [S12] for more details of the method), for each participant separately and the best model (least error) was used for inference on model parameters. The code can be made available upon request. This non-hierarchical DDM (NHDDM) allowed us to validate the HDDM method described above by specifying exact experimental manipulations (e.g. slope in drift rate) and furthermore allowed second (group) level regression analyses with neural parameters, which cannot be computed at the single trial level (inter-site-phase-clustering; see below). We compared results of the HDDM and NHDDM by assessing parameter estimates at the group and individual subject level and assessed NHDDM fits by computing quantile probability plots (figure S2).

Finally, we validated the HDDM by fitting it to a simulated dataset. The simulation was based on estimated parameters derived from NHDDM for a representative patient (patient with parameters which were closest to the group mean). We simulated a dataset consisting of 300 trials per condition based on the number of trials for the whole group. We used conditions with low, medium and high unidirectional coherence in task A and medium unidirectional coherence in task B (i.e. 1200 trials) in order to model a manipulation of drift rate slope (low and high relative to medium unidirectional coherence) and threshold (medium unidirectional coherence in task B relative to medium unidirectional coherence in task A). We then assessed whether HDDM could successfully recover the true condition-specific manipulations of parameters based on the simulated dataset. To this end, we computed model evidence (DIC) for the true model compared to alternative models comprising different possible combinations of drift rate and threshold modulations during the different conditions. Furthermore, we assessed whether the true parameter manipulations could be recovered (effects on drift rate and threshold) and compared the predicted with the observed RT distribution. This analysis showed that HDDM successfully recovered the true parameter manipulations, which is shown in figure S3.

After verifying assumptions of the HDDM applied in this study, we then entered trial-by-trial STN-LFPs (z-scored) into the HDDM to test whether fluctuations in STN activity modulated decision-making parameters. We z-scored single trial estimates of STN power by subtracting the mean and dividing by the standard deviation separately for task A and task B. To further assess intercept issues, we repeated the HDDM analysis using non-z-scored data, which yielded identical results. Thus, regression coefficients between STN-LFPs and the decision parameter were estimated within the same hierarchical model, which was used to estimate the parameters themselves. For example, the model postulating that decision threshold a on trial x was not only modulated by Task but also STN-LFO (and their interaction) was defined by the regression: $a(x) = \beta_0 + \beta_1 Task(x) + \beta_2 LFO(x) + \beta_3 Task(x)*LFO(x)$ Regressing across trials allowed inferences on the extent to which threshold changes with STN activity [S8]. We created four a-priori defined models which differed regarding the frequency band of STN power (LFO vs. Beta) and the decision parameter which was modulated (threshold vs. drift rate), see Fig. 2B. These models were then compared using their DIC (relative to the model not containing any neural data). The best model was used for inferences on model parameters using Bayesian hypothesis testing, i.e. according to their posterior probability densities (see above). Posteriors of regression coefficients for trial-wise regressors were estimated only at the group level to address potential collinearity among model parameters, for regularizing parameter estimates and to prevent parameter explosion [S8, S9].

Analysis of FCz-STN inter-site-phase-clustering and regression analyses

Models of basal ganglia function during decision-making postulate that decision thresholds can be adjusted through activation of a connection between mPFC and STN in case of choice uncertainty or decision conflict [S2, S5, S9, S13-15]. To test this hypothesis, we aimed to assess whether mPFC-STN connectivity predicted adjustment of decision-thresholds and whether this relationship differed between low and high levels of cautiousness. However, connectivity parameters are more robust when averaged across trials compared to single-trial parameter estimates. Therefore, we computed estimates of inter-site-phase-clustering (IPC), a phase-based measure of connectivity, between FCz and STN for each subject and task and tested whether this measure predicted inter-individual differences in decision-thresholds. We only analyzed IPC between FCz and STN, because our previous studies have shown that task-related changes in cortico-STN connectivity were specific for the FCz-STN connection and were not observed between Pz and STN [S2, S3]. Please note that while we cannot discount more lateral prefrontal areas contributing to the signal at FCz due to the limited coverage in this study, previous studies have provided converging evidences that conflict-related LFO at FCz are generated in the mPFC [S5, S9, S16]. IPC was used as a measure of the extent to which oscillations in the mPFC and STN were phase-locked before the response [S2]. Analysis was done separately for task A and task B. Raw data were band-pass filtered between 2 and 8 Hz, and power and phase were computed for each STN channel and the FCz recording using the Hilbert transform. Then the magnitude of the average

phase difference between the STN-LFP and EEG signal were calculated at each time point, averaged across trials and a sliding window (1.6 s) was applied for integrating over time [S2]. For specifically computing the pre-response change in IPC values were averaged across the time window from -3 to 0 and divided by the mean peri-response (-5 to + 2s) value. This was done for each STN-channel and FCz recording separately and then averaged across STN channels for each participant resulting in one IPC value for each patient and task, which was used for regression analyses.

In order to test whether inter-individual differences in modulation of IPC between FCz and STN predicted differences in adjustments of decision thresholds we applied linear regression analyses. We used the difference in FCz-STN IPC between task B and task A (positive values indicating increased phase coupling) as predictor and the corresponding change in threshold estimates derived from the NHDDM as dependent variable. Since elevated decision-thresholds are thought to improve the ability to control erroneous responses, a second regression with the same predictor was conducted using the change in accuracy during trials with an initial bidirectional coherence (see results) as dependent variable. To account for possible confounding effects of differences in drift rate we additionally repeated these regression analyses partialing out drift rate estimates (partial regression). Note that we did not use the parameter estimates from the HDDM, because the hierarchical design violates the assumption of independence of observations. Prior to conducting regression analyses we ensured that assumptions of linear regression were not violated including independence of observations, lack of outliers (> 3*SD), and approximate normal distribution of residuals. The significance threshold for was set to 0.05 two-tailed.

Supplemental References:

S1. Huang, Y.T., Georgiev, D., Foltynie, T., Limousin, P., Speekenbrink, M., and Jahanshahi, M. (2015). Different effects of dopaminergic medication on perceptual decision-making in Parkinson's disease as a function of task difficulty and speed-accuracy instructions. Neuropsychologia *75*, 577-587.

S2. Zavala, B.A., Tan, H., Little, S., Ashkan, K., Hariz, M., Foltynie, T., Zrinzo, L., Zaghloul, K.A., and Brown, P. (2014). Midline frontal cortex low-frequency activity drives subthalamic nucleus oscillations during conflict. J Neurosci *34*, 7322-7333.

S3. Zavala, B.A., Tan, H., Little, S., Ashkan, K., Hariz, M., Foltynie, T., Zrinzo, L., Zaghloul, K.A., and Brown, P. (2015). Decisions made with less evidence involve higher levels of cortico-STN theta band synchrony. Journal of Cognitive Neuroscience *in revision*.

S4. Peirce, J.W. (2007). PsychoPy--Psychophysics software in Python. J Neurosci Methods *162*, 8-13.

S5. Cavanagh, J.F., Wiecki, T.V., Cohen, M.X., Figueroa, C.M., Samanta, J., Sherman, S.J., and Frank, M.J. (2011). Subthalamic nucleus stimulation reverses mediofrontal influence over decision threshold. Nat Neurosci *14*, 1462-1467.

S6. Zavala, B., Brittain, J.S., Jenkinson, N., Ashkan, K., Foltynie, T., Limousin, P., Zrinzo, L., Green, A.L., Aziz, T., Zaghloul, K., et al. (2013). Subthalamic nucleus local field potential activity during the Eriksen flanker task reveals a novel role for theta phase during conflict monitoring. J Neurosci *33*, 14758-14766.

S7. Hammond, C., Bergman, H., and Brown, P. (2007). Pathological synchronization in Parkinson's disease: networks, models and treatments. Trends Neurosci *30*, 357-364.

S8. Wiecki, T.V., Sofer, I., and Frank, M.J. (2013). HDDM: Hierarchical Bayesian estimation of the Drift-Diffusion Model in Python. Front Neuroinform *7*, 14.

S9. Frank, M.J., Gagne, C., Nyhus, E., Masters, S., Wiecki, T.V., Cavanagh, J.F., and Badre, D. (2015). fMRI and EEG predictors of dynamic decision parameters during human reinforcement learning. J Neurosci *35*, 485-494.

S10. Ratcliff, R., and McKoon, G. (2008). The diffusion decision model: theory and data for two-choice decision tasks. Neural Comput *20*, 873-922.

S11. Zhang, J., and Rowe, J.B. (2014). Dissociable mechanisms of speed-accuracy tradeoff during visual perceptual learning are revealed by a hierarchical drift-diffusion model. Front Neurosci *8*, 69.

S12. Bogacz, R., and Cohen, J.D. (2004). Parameterization of connectionist models. Behav Res Methods Instrum Comput *36*, 732-741.

S13. Frank, M.J., Samanta, J., Moustafa, A.A., and Sherman, S.J. (2007). Hold your horses: impulsivity, deep brain stimulation, and medication in parkinsonism. Science *318*, 1309-1312.

S14. Ridderinkhof, K.R., Forstmann, B.U., Wylie, S.A., Burle, B., and van den Wildenberg, W.P.M. (2011). Neurocognitive mechanisms of action control: resisting the call of the Sirens. . WIREs Cogn Sci *2*, 174–192.

S15. Wiecki, T.V., and Frank, M.J. (2013). A computational model of inhibitory control in frontal cortex and basal ganglia. Psychol Rev *120*, 329-355.

S16. Cohen, M.X. (2014). A neural microcircuit for cognitive conflict detection and signaling. Trends Neurosci *37*, 480-490.
